# Supplementary material for: General technoeconomic analysis for electrochemical coproduction coupling carbon dioxide reduction with organic oxidation
Source: Nat Commun. 2019 Nov 15;10:5193. doi: 10.1038/s41467-019-12744-y (PMC6858374; doi:10.1038/s41467-019-12744-y)
Supplement: Supplementary file 1 — Supplementary Information [file 41467_2019_12744_MOESM1_ESM.docx]

**Supplementary Information for**

**General technoeconomic analysis for electrochemical coproduction coupling carbon dioxide reduction with organic oxidation**

*Na et al.*

**Supplementary Methods**

**Cathodic reaction: electrocatalytic reduction of CO_2_.** Regarding reduction, electrochemical CO_2_RR and the H_2_ evolution reaction (HER) have attracted considerable attention because these reactions are potential methods to produce useful chemicals and clean fuels from abundant carbon sources, water, and renewable electricity.^1-6^ The target products include hydrogen, carbon monoxide, methane, ethylene, and ethanol.^7-11^ To assess the economic feasibility of the production of these products in electrochemical coproduction systems, the FE, partial current density, and cell potential, which are directly related to productivity, must be considered during TEA. Based on these criteria, the target products were divided into three categories, shown in Table 1, and their essential information, including the half-cell reaction equation, standard reduction potential (E^0^) overpotential (η), and market price, is listed in Supplementary Table 2. First, H_2_, CO, formic acid, and ethylene have a high FE and high partial current density.^12-21^ These compounds have an FE of > 70% and a current density of > 100 mA cm^-2^. The HER in particular has a low overpotential, close to 0 V vs. RHE, and the FE is close to 100%. For this reason, many related studies have been reported, and empirical studies on mass production are also actively being carried out. Substantially, these products are fundamental precursors of many chemical products. Furthermore, H_2_ and formic acid are used as energy carriers without self-discharge, which can be used to generate electricity using fuel cell systems.^22-24^ The second group includes ethanol and *n*-propanol, which are liquid-type products and fundamental organic chemicals. Although these products have a low FE and partial current density, they have relatively high market prices and are easy to transport, and therefore their economic feasibility can be improved by increasing the partial current density.^25^ The third group has an FE of < 1% and a partial current density of < 0.5 mA cm^-2^ in the CO_2_RR process.^26^ Though these products are produced in small quantities, they are fundamental chemical substances that can be used to produce various valuable chemical products.

**Anodic reaction: CO_2_ reduction paired with organic oxidation.** The oxidation reaction in electrochemical coproduction systems generally uses the O_2_ evolution reaction (OER). However, the OER is not an attractive reaction in terms of energy because oxygen is produced at high oxidative potential (normally> 1.48 vs. RHE, Supplementary Table 3). In addition, O_2_ is a low-value product compared to other value-added organics from the cathode. Therefore, effective alternative anodic half-reactions are highly desirable to decrease the overall energy consumption.

Recently, the electrochemical organic oxidation reaction (OOR) has emerged as an effective alternative for coupling with the HER. HER-OOR coupling is an attractive option not only because of economically high value products but because of potentially low overall cell voltage (Figure 1b).^27-30^ For example, HMF oxidation reaction has 1.25 V onset potential and can achieve 20 mA/cm^2^ current density at 1.32 V. The methanol oxidation reaction to formic acid occurs even lower potential (> 0.6 V) than the standard reduction potential of oxygen. The overall cell potential adopted in this study for each coupling reaction can be calculated using Supplementary Table 2 and 3. Note that the overall cell voltages of organic compounds vary depending on the functional group to be oxidized and on electrolysis conditions such as electrolyte, pH, and electrode materials. Thus, we analyze the influence of the overall cell potential on the economic potential through global sensitivity analysis. In this regard, significant energy savings are expected when OOR is combined with CO_2_RR and the coproduction of value-added organics in both electrodes is realized. A number of chemicals have been considered as potential candidates for OOR. Based on the source of raw material, we divided these candidates into three categories, and their essential information is shown in Table 1 and Supplementary Table 3. The products or organic raw materials in the first group (denoted as Group IV), such as methanol, ethanol, n-propanol, and ethylene glycol, are also cathode products. Formaldehyde and formic acid are obtained from the oxidation of methanol, and ethanol can be oxidized to acetaldehyde, acetic acid, and ethyl acetate. Acrylic acid and lactic acid are synthesized by the oxidation of n-propanol, and ethylene glycol is oxidized to glycolic acid and oxalic acid. The second group (denoted as Group V) is biomass intermediates, which includes 2-furoic acid and 2,5-furandicarboxylic acid (FDCA). 2-Furoic acid is oxidized from furfuryl alcohol or furfural. FDCA is synthesized in the oxidation of 5-hydroxymethylfurfural (HMF), which is a renewable carbon source obtained from biomass.^31^ The resulting product, FDCA, can serve as a monomer to synthesize poly(ethylene 2,5-furandicarboxylate) (PEF), which has attracted considerable attention as a renewable polymer capable of replacing polyethylene terephthalate (PET).^32^ The third group includes oxygen, aldehyde, carboxylic acid, and ketone from the oxidation of water and alcohol.

A very recent work demonstrated the combination of electrolytic CO_2_RR and OOR.^33^ In this proof-of-concept study, four different types of cells for CO_2_RR paired with OOR.

**Electrolyzer devices.** The reactor design in electrochemical processes has a profound effect on the FE and partial current density. However, electrochemical coproduction has no standardized device or well-established configuration, making economic feasibility analysis difficult. As shown in Supplementary Fig. 1, several devices have been proposed for water splitting and CO_2_RR, according to the presence of an ion-exchange membrane and the raw material supply method. Kenis *et al*.^34-37^ introduced a CO_2_RR device comprising a microfluidic flow cell modified from a microfluidic fuel cell. In this device, the anode and cathode active surfaces are separated by an aqueous electrolyte channel. In addition, the reference electrode inserted in the electrolyte flow field measures the applied potential on each electrode. Microfluidic flow cells, however, may not be applicable in electrochemical coproduction systems because the cathode and anode products are difficult to separate from the electrolyte solution. The separation of cathode and anode products often requires the introduction of an ion-exchange membrane. A typical device concept with an ion-exchange membrane is shown in Supplementary Fig. 1b.^38-40^ This configuration consists of two flow channels, and the anode and cathode are separated by an ion-exchange membrane. The electrocatalysts are coated on the gas diffusion layer (GDL) and placed in the separated flow field. The electrolyte or gas-saturated electrolyte is supplied directly to the anode and cathode sides. In contrast to microfluidic flow cells, this system is relatively easy to pressurize, and the device can be industrialized by constructing large stack systems.^41,42^ However, the low gas solubility in the electrolyte limits the FE and partial current density. For this reason, the direct gas-phase feeding method (Supplementary Fig. 1c) has recently been intensively researched.^43^ Indeed, devices with gas-phase feeding exhibit improved partial current density compared to that under aqueous-phase conditions. The device concept in Supplementary Fig. 1d has a liquid buffer layer between the cathode catalyst layer and the ion-exchange membrane. The gas-phase feed is directly supplied to the cathode side. The advantage of this kind of system is that it restricts the proton (H^+^) concentration and can suppress the competitive HER at oxidation electrodes.^44,45^ In addition, the controlled hydrophobicity of the cathode can increase the FE and partial current density by reducing the required diffusion length of gas resources and accelerating mass transfer.^12,46,47^

**Brief summary of previous economic analysis.** Despite the great interest and active research regarding coproduction methods coupling electrochemical CO_2_RR with OOR,^29-31,33,48-61^ there has been no systematic analysis of the economic viability of the process. Since research on the TEA of CO_2_RR or HER plants coupled with OER has been conducted, it is necessary to review the chemicals that have been analyzed and claimed to be economically feasible. The economic metrics and economic analysis methodology also have to be reviewed. A brief summary of previous TEA research and the proposed product chemicals is shown in Supplementary Table 6. TEAs of solar-to-fuel (STF) technology have evaluated economic feasibility using energy-based metrics (energy return on energy invested (EROEI); energy payback time (EPBT); and energy incorporation efficiency (EIE), which compares the total primary energy resources consumed to the higher heating value (HHV)).^62,63^ This type of economic evaluation does not include the capital cost of the entire process and cannot readily reflect different utility costs. Although economic analysis based on energy metrics can be carried out easily, nonfuel chemicals cannot be evaluated in terms of energy. In addition, when the energy-based metrics are not necessarily the same as the cost-based metrics, further evaluation may be needed for more rigorous technology development. To perform a more reliable TEA, recent studies have employed economic metrics based on net present value (NPV). In particular, LCCs that result in an NPV of 0 have been actively used for comparison with the present market price.^64-67^ This approach has recently drawn increasing attention because several cost-related factors for electrochemical plants have been released in the DOE H2A central hydrogen production model.^68^ Even if we do not know the exact market price of a product, the LCC can be estimated, approximately reflecting the economic feasibility. In particular, chemicals that are not currently in mass production, such as FDCA, have no market price. Thus, we can estimate the production cost from the LCC. From the studies identified above, the most promising products in CO_2_RR coupled with OER are carbon monoxide and formic acid, based on the present maximum current density and FE. Ethylene glycol and propanol are also promising if high efficiency can be achieved through catalyst and device development.

Although the above papers provide relatively useful information through a wide variety of approaches, a TEA study related to electrochemical coproduction has not been performed because of the following limitations: 1) It is difficult to accurately estimate profit because of uncertain product sales. This uncertainty is caused by the unavailability and uncertainty of market sales of some products and uncertainty propagation through external factors (e.g., the quantity of solar radiation). 2) The precise total capital investment (TCI) is difficult to evaluate through a conceptual process design of the electrochemical process. In particular, most technoeconomic studies do not explicitly address separation systems; instead, the approaches used include assuming universal distillation,^64^ using Sherwood plots,^69^ disregarding separation,^70^ and calculating the theoretical free energy required for separation.^62^ Such approaches may underestimate both capital costs and operation costs. 3) Depending on the products, different types of optimal process designs are required; this limitation has not yet been considered. 4) It is difficult to perform a qualitative analysis of physical feasibility.

**Supplementary Notes**

**Process modeling and simulations for CO/2-furoic acid case.** To understand the procedure of complicated technoeconomic analysis, here we provided the example case as electrochemical CO_2_ reduction to CO for a cathode and furfural oxidation to 2-furoic acid for an anode. Parameters were set to the base case (Supplementary Table 4).

1. **Process systems modeling based on superstructure**

Since the phase of cathode product and anode product are gas and liquid, respectively, and CO cannot be oxidized (which means cascade structure cannot be applied), the superstructure G can be reduced by Algorithm 1 (Supplementary Fig. 10a and b). We employed three inlet streams which are CO_2_, furfural, and electrolyte make-up stream. Two product outlet streams (CO and 2-furoic acid), two byproduct outlet streams (H_2_ and O_2_), and three purge streams were also considered (Supplementary Fig. 10c). In the case of cathode part, CO was the gas product which needs the gas/liquid separation (flash drum) for electrolyte separation and two step gas/gas separations (CCS and pressure swing adsorption), for CO_2_ separation and H_­2_ separation (Supplementary Fig. 10c). For the anode part, gas/liquid separation for O_2_ and two step liquid/liquid separations (extraction with distillation for solvent regeneration) for electrolyte with furfural and regeneration of extraction solvent (Supplementary Fig. 10c).

1. **Simulation**

- PV-EC calculation

Actual amount of produced electricity from photovoltaic system:

${\bar{\text{E}}}_{\text{PV}}\text{=}\text{E}_{\text{farm}}\text{η}_{\text{CF}}\text{=40 MW}\text{×0.2×}\text{10}^{\text{6}}\frac{\text{W}}{\text{MW}}\text{ }\text{=8.00}\text{×}\text{10}^{\text{6}} W$*.*

Total required electrolyzer cell area A_cell_:

$\text{A}_{\text{cell}}\text{=}\frac{{\bar{\text{E}}}_{\text{PV}}}{\text{(}\text{E}_{\text{anode}}^{\text{0}}\text{+}\text{η}_{\text{anode}}-\text{E}_{\text{cathode}}^{\text{0}}\text{+}\text{η}_{\text{cathode}}\text{)}\text{×}\text{CD}}\text{=}\frac{\text{8.00}\text{×}\text{10}^{\text{6}}\text{ W}}{\left( \text{-1.27+2.7-(-0.1)+}\text{0.9} \right)\text{ V × }\text{500}\frac{\text{mA}}{\text{cm}^{\text{2}}}\text{ }}\text{×}\text{10}^{\text{3}}\frac{\text{mA}}{\text{A}}\text{×}\text{10}^{\text{-4}}\frac{\text{m}^{\text{2}}}{\text{c}\text{m}^{\text{2}}}$ = 658 m^2^.

The production rate of CO:

${\dot{\text{n}}}_{\text{cathode}}^{\text{out}}\text{=}\frac{\text{CD}\text{×}\text{F}\text{E}_{\text{cathode}}{\text{×}\text{A}}_{\text{cell}}}{\text{F}\text{×}\text{z}_{\text{cathode}}}\text{=}\frac{\text{500}\frac{\text{mA}}{\text{cm}^{\text{2}}}\text{×0.9×}\text{658}\text{ }\text{m}^{\text{2}}}{\text{96485}\frac{\text{C}}{\text{mol}}\text{×2}}\text{×}\text{10}^{\text{-3}}\frac{\text{A}}{\text{mA}}\text{×}\text{10}^{\text{4}}\frac{\text{c}\text{m}^{\text{2}}}{\text{m}^{\text{2}}}\text{=15.35}\frac{\text{mol}}{\text{sec}}$*.*

The production rate of 2-furoic acid:

${\dot{\text{n}}}_{\text{anode}}^{\text{out}}\text{=}\frac{\text{CD}\text{×}\text{F}\text{E}_{\text{anode}}{\text{×}\text{A}}_{\text{cell}}}{\text{F}\text{×}\text{z}_{\text{anode}}}\text{=}\frac{\text{500}\frac{\text{mA}}{\text{cm}^{\text{2}}}\text{×0.9×}\text{658}\text{ }\text{m}^{\text{2}}}{\text{96485}\frac{\text{C}}{\text{mol}}\text{×2}}\text{×}\text{10}^{\text{-3}}\frac{\text{A}}{\text{mA}}\text{×}\text{10}^{\text{4}}\frac{\text{c}\text{m}^{\text{2}}}{\text{m}^{\text{2}}}\text{=15.35}\frac{\text{mol}}{\text{sec}}$*.*

- Heat and mass balance using Aspen Plus process simulator

Using the production rate of CO and 2-furoic acid, stoichiometric numerical simulation was performed using commercial process simulator Aspen Plus. Inlet flowrate of CO_2_ was set by 110% of the required flowrate calculated by CO production rate. 0.1 M KHCO_3_ and CO_2_(aq) 33 mM (VLE of 1 bar CO_2_(g) with Henry’s law) electrolyte was considered and inlet flowrate was set to match the CO_2_ inlet flowrate using design specification in simulator. Inlet flow rate of organic raw materials for anodic oxidation was set by multiplying 110% of the required flowrate calculated by product production rate. Operation pressure and temperature were set to 101325 Pa and 25^o^C, respectively.

1. **Technoeconomic analysis**

The procedure for the technoeconomic evaluation using the values ​​obtained by the CO/2-furoic acid case study simulation is as follows. Base framework was referred from Seider et al.^71^

- Capital cost

| Total bare-module costs for equipments | |  |  |
| --- | --- | --- | --- |
|  | Electrolyzer |  | $12,543,176 |
|  | Flash |  | $540,358 |
|  | Compressor |  | $1,363,197 |
|  | Distillation |  | $437,153 |
|  | Extraction |  | $4,466,735 |
|  | PSA |  | $140,507 |
|  | HX |  | $7,465 |
| Total bare-module costs for computers and software | |  | $20,000 |
| **Total bare-module investment, TBM** | |  | **$19,518,592** |
| Cost of site preparation | | 0.1×TBM | $1,951,859 |
| **Total of direct permanent investment, DPI** | |  | **$21,470,451** |
| Cost of contingencies and contractor's fee | | 0.15×DPI | $3,220,568 |
| **Total depreciable capital, TDC** | |  | **$24,691,019** |
| Cost of land | | 0.02×TDC | $493,820 |
| Cost of Plant startup | | 0.02×TDC | $493,820 |
| **Total permanent investment, TPI** | |  | **$25,678,660** |

- Operating cost and the cost sheet

| **Cost Factor** | | | **Annualized Cost ($)** |
| --- | --- | --- | --- |
| **Feedstocks** | |  |  |
|  | Electrolyte | | $41,606,293 |
|  | CO_2_ |  | $1,179,368 |
|  | Furfural |  | $64,540,110 |
| **Utilities** |  |  |  |
|  | Electricity |  | $9,319,589 |
|  | Steam |  | $494,031 |
|  | Regrigeration | | $125,545 |
|  | Wastewater treatment | | $492,003 |
|  | CO2 capture cost | | $114,618 |
| **Operations (labor-related) (O)** | | |  |
|  | Direct wages and benefits (DW&B) | | $2,800,000 |
|  | Direct salaries and benefits | | 15% of DW&B |
|  | Operating supplies and services | | 6% of DW&B |
|  | Technical assistance to manufacturing | | $200,000 |
|  | Control laboratory | | $216,667 |
| **Maintenance (M)** | |  |  |
|  | Wages and benefits (MW&B) | | $864,186 |
|  | Salaries and benefits | | 25% of MW&B |
|  | Materials and services | | 100% of MW&B |
|  | Maintenance overhead | | 5% of MW&B |
| **Operating overhead** | | |  |
|  | General plant overhead | | 7.1% of M&O-SW&B |
|  | Mechanical department services | | 2.4% of M&O-SW&B |
|  | Emplyee relations department | | 5.9% of M&O-SW&B |
|  | Business services | | 7.4% of M&O-SW&B |
| **Property taxes and insurance** | | | 2% of TDC |
| **Depreciation** | |  |  |
|  | Direct plant | | 8% of (TDC-1.18alloc) |
|  | Allocated plant | | 6% of 1.18alloc |
| **COST OF MANUFACTURES (COM)** | | | **$115,113,677** |
| **General Expenses** | |  |  |
|  | Selling (or transfer) expense | | 3% (1%) of sales |
|  | Direct research | | 4.8% of sales |
|  | Allocated research | | 0.5% of sales |
|  | Administrative expense | | 2.0% of sales |
|  | Management incentive compensation | | 1.25% of sales |
| **TOTAL GENERAL EXPENSES (GE)** | | | **$46,999,371** |
| **TOTAL PRODUCTION COST (C)** | | | **$162,113,047** |
| Sales |  |  |  |
|  | Cathode products | | $7,309,953 |
|  | Anode products | | $304,410,756 |
| **TOTAL SALES (S)** | |  | **$311,720,709** |

- Cash flow and the levelized cost

Cash flow analysis was performed with 15 years plant life, 2 years plant construction period, 5 years clas life MACRS depreciation, 15% nominal interest rate, and 38.9% income tax rate to calculate NPV (Eqns 9-11). The levelized cost of each product was calculated by NPV equals to zero. The recommended price, which lay on the levelized cost line, was calculated to equal the ratio between the market price and the recommended price as,

$\frac{\text{Market Pric}\text{e}_{\text{2-furoic acid}}}{\text{Market Pric}\text{e}_{\text{CO}}}\text{=}\frac{\text{Recommended pric}\text{e}_{\text{2-furoic acid}}}{\text{Recommended pric}\text{e}_{\text{CO}}}$.

In case of CO/2-furoic case, the levelized cost can be formed at a lower price than market price, which guarantees the economic feasibility (Supplementary Fig. 11a). In the capital cost, the electrolyzer occupies 64.3% and the extraction system to separate 2-furoic acid accounts for 22.9%. Since, it is an electrochemical process that uses a large amount of electricity, electricity cost contributes to 88.4% to overall operating cost (Supplementary Fig. 11b). Interestingly, the cost of operating the treatment system for organic waste is 4.7% which is higher than expected (Supplementary Fig. 11b). It is expected to be in surplus from the third year of investment and is worth about $ 503 million NPV in the 15^th^ year of plant life (Supplementary Fig. 11c and Supplementary Table 10).

**Supplementary Tables**

**Supplementary Table 1** Electrolysis conditions of cathodic/anodic products in the parallel process

| **Product** | **Catalyst** | **pH** | **Temperature (℃)** | **Electrolyte** | **Ref.** |
| --- | --- | --- | --- | --- | --- |
| **Cathode products** | | | | | |
| H_2_ | Pt, MoS_2_ | 0 - 1 | 25 - 80 | H_2_SO_4_ / HClO_4_ Solution, PEM | ^16,72-76^ |
|  | Pt | 13 - 14 | 25 - 80 | KOH Solution, AEM |  |
| Syngas | Ag | 6 - 7 | 25 | KHCO_3_ Solution, AEM | ^77-80^ |
| CO | Ag | 6 - 7 | 25 | KHCO_3_ Solution, AEM | ^81-83^ |
| Formate | Sn, SnO_2_ | 6 - 7 | 25 | KHCO_3_ Solution, AEM | ^84-86^ |
| Methanol | Cu | 6 - 7 | 25 | KHCO_3_ Solution, AEM | ^87,88^ |
| Methane | Cu | 6 - 7 | 25 | KHCO_3_ Solution, AEM | ^87,89^ |
| Ethylene | Cu | 6 - 7 | 25 | KHCO_3_ Solution, AEM | ^90-92^ |
| Ethanol | Cu, CuAg | 6 - 7 | 25 | KHCO_3_ Solution, AEM | ^93-95^ |
| n-Propanol | Cu, CuZnO | 6 - 7 | 25 | KHCO_3_ Solution, AEM | ^87,93,96^ |
| Acetaldehyde | Cu | 6 - 7 | 25 | KHCO_3_ Solution, AEM | ^87,97^ |
| Glyoxal | Cu | 6 - 7 | 25 | KHCO_3_ Solution, AEM | ^87,98^ |
| Acetone | Cu | 6 - 7 | 25 | KHCO_3_ Solution, AEM | ^87,99^ |
| Acetate | Cu | 6 - 7 | 25 | KHCO_3_ Solution, AEM | ^87,100^ |
| Ethylene glycol | Cu, Au | 6 - 7 | 25 | KHCO_3_ Solution, AEM | ^87,101^ |
| **Anode Products** | | | | | |
| O_2_ | Ir, Ru | 0 - 1 | 25 - 80 | H_2_SO_4_ / HClO_4_ Solution, PEM | ^102,103^ |
|  | Ni, Fe, Co | 13 - 14 | 25 - 80 | KOH Solution, AEM |  |
| Hydrogen peroxide | Pt | 0 - 1 | 25 | H_2_SO_4_ / HClO_4_ Solution, PEM | ^104,105^ |
|  |  | 13 - 14 | 25 | KOH / NaOH Solution |  |
| Acetaldehyde | Ti-Pt | 0 - 1 | 25 - 80 | H_2_SO_4_ / HClO_4_ Solution | ^106,107^ |
| Acetic acid | Pd-TiO_2_,  FeOOH | 0 - 1 | 25 - 80 | H_2_SO_4_ / HClO_4_ Solution | ^106-108^ |
| Ethyl acetate | Co_3_O_4_, PdCu | 13 - 14 | 25 | KOH Solution | ^109^ |
| Acrylic acid | Pt, Au, Pd-CeO_2_ | 0 - 1 | 25 | HClO_4_ Solution | ^110^ |
| Lactic acid | Au, AuPt, Co | 13 - 14 | 25 | KOH Solution, AEM | ^111^ |
| Benzaldehyde | Cu-Co-N | - | 45 | Ionic Liquid [Bmin][BF_4_] | ^112,113^ |
| Benzoic acid | Ni | 0 - 1 | 25 - 90 | H_2_SO_4_ Solution | ^114,115^ |
| 2-Furoic acid | Ni_3_S_2_ | 9.4 | 25 - 80 | NaHCO_3_/Na_2_CO_3_ | ^116,117^ |
|  | Ni_2_P/Ni | 13 - 14 | 30 | KOH Solution |  |
| 2,5-Furandicarboxylic acid  (FDCA) | MnO_2_ | 1 | 60 | H_2_SO_4_ Solution | ^118,119^ |
|  | NiFe | 13 - 14 | 25 | KOH Solution |  |
| 4-Methoxybenzaldehyde | Graphite | - | 25 | TEMPO Solution | ^120^ |
| Acetophenone | - | - | 25 | CH_2_Cl_2_ Solution | ^121^ |
| Acetone | NiS_2_ | 13 - 14 | 25 | KOH Solution | ^122,123^ |
| Phenoxyacetic acid | Ni | 13 - 14 | 25 | NaOH Solution | ^124^ |
| Formaldehyde | - | 0 - 1 | 25 | HClO_4_ Solution | ^125,126^ |
| Formic acid | Pt | 0 - 1 | 25 | H_2_SO_4_ Solution | ^127,128^ |
| Glycolic acid | Pd-TiO_2_ | 13 - 14 | 25 | KOH Solution | ^129^ |
| Oxalic acid | FeCoNi | 13 - 14 | 25 | KOH Solution | ^129^ |

* Mt: Mega Tonne, * PEM: Proton Exchange Membrane, * AEM: Anion Exchange Membrane

**Supplementary Table 2** Essential information of the reduction products for the cathode

| **Product** | **Half-cell reaction** | $\mathbf{E}^{\mathbf{0}}$**(V)** | $\boldsymbol{\eta}$**(V)** | **Market price ($/kg)** |
| --- | --- | --- | --- | --- |
| Hydrogen | $\text{2}\text{H}^{\text{+}}\text{+2}\text{e}^{\text{-}}\text{ → }\text{H}_{\text{2}}$ | 0 | 0.05^72^ | 1.39^130^ |
| Carbon monoxide | $\text{CO}_{\text{2}}\text{+2}\text{H}^{\text{+}}\text{+2}\text{e}^{\text{-}}\text{ → CO+}\text{H}_{\text{2}}\text{O}$ | -0.1 | 0.9^81^ | 0.6^131^ |
| Formic acid | $\text{CO}_{\text{2}}\text{+2}\text{H}^{\text{+}}\text{+2}\text{e}^{\text{-}}\text{ → Formic acid}$ | -0.02 | 1.01^84^ | 0.97-1.08^132^ |
| Methanol | $\text{CO}_{\text{2}}\text{+6}\text{H}^{\text{+}}\text{+6}\text{e}^{\text{-}}\text{ → Methanol+}\text{H}_{\text{2}}\text{O}$ | 0.03 | 0.84^133^ | 0.34-0.49^134^ |
| Methane | $\text{CO}_{\text{2}}\text{+8}\text{H}^{\text{+}}\text{+8}\text{e}^{\text{-}}\text{ → Methane+2}\text{H}_{\text{2}}\text{O}$ | 0.17 | 1.34^87^ | 0.21^135^ |
| Ethylene | $\text{2}\text{CO}_{\text{2}}\text{+12}\text{H}^{\text{+}}\text{+12}\text{e}^{\text{-}}\text{ → Ethylene+4}\text{H}_{\text{2}}\text{O}$ | 0.08 | 0.62^136^ | 0.35-0.71^137^ |
| Ethanol | $\text{2}\text{CO}_{\text{2}}\text{+12}\text{H}^{\text{+}}\text{+12}\text{e}^{\text{-}}\text{ → Ethanol+3}\text{H}_{\text{2}}\text{O}$ | 0.09 | 0.95^138^ | 0.61-0.75^139^ |
| *n*-Propanol | $\text{3}\text{CO}_{\text{2}}\text{+18}\text{H}^{\text{+}}\text{+18}\text{e}^{\text{-}}\text{ → }\text{n}\text{-Propanol+5}\text{H}_{\text{2}}\text{O}$ | 0.1 | 0.84^138^ | 1.43^131^ |
| Acetaldehyde | $\text{2}\text{CO}_{\text{2}}\text{+10}\text{H}^{\text{+}}\text{+10}\text{e}^{\text{-}}\text{ → Acetaldehyde+3}\text{H}_{\text{2}}\text{O}$ | 0.05 | 1.09^87^ | 0.93-1.14^140^ |
| Hydroxyacetone | $\text{3}\text{CO}_{\text{2}}\text{+14}\text{H}^{\text{+}}\text{+14}\text{e}^{\text{-}}\text{ → Hydroxyacetone+4}\text{H}_{\text{2}}\text{O}$ | 0.46 | 1.47^87^ | 0.79^141^ |
| Acetone | $\text{3}\text{CO}_{\text{2}}\text{+16}\text{H}^{\text{+}}\text{+16}\text{e}^{\text{-}}\text{ → Acetone+5}\text{H}_{\text{2}}\text{O}$ | -0.14 | 0.91^87^ | 0.9-1.28^142^ |
| Acetic acid | $\text{2}\text{CO}_{\text{2}}\text{+8}\text{H}^{\text{+}}\text{+8}\text{e}^{\text{-}}\text{ → Acetic acid+2}\text{H}_{\text{2}}\text{O}$ | -0.26 | 0.79^87^ | 0.68-0.92^143^ |
| Allyl alcohol | $\text{3}\text{CO}_{\text{2}}\text{+16}\text{H}^{\text{+}}\text{+16}\text{e}^{\text{-}}\text{ → Allyl alcohol+5}\text{H}_{\text{2}}\text{O}$ | 0.11 | 1.12^87^ | 2.42^144^ |
| Glycolaldehyde | $\text{2}\text{CO}_{\text{2}}\text{+8}\text{H}^{\text{+}}\text{+8}\text{e}^{\text{-}}\text{ → Glycolaldehyde+2}\text{H}_{\text{2}}\text{O}$ | -0.03 | 1.1^87^ | 1.06^141^ |
| Propionaldehyde | $\text{3}\text{CO}_{\text{2}}\text{+16}\text{H}^{\text{+}}\text{+16}\text{e}^{\text{-}}\text{ → Propionaldehyde+5}\text{H}_{\text{2}}\text{O}$ | 0.14 | 1.14^87^ | 0.96^145^ |
| Ethylene glycol | $\text{2}\text{CO}_{\text{2}}\text{+10}\text{H}^{\text{+}}\text{+10}\text{e}^{\text{-}}\text{ → Ethylene glycol+2}\text{H}_{\text{2}}\text{O}$ | 0.2 | 0.78^146^ | 0.83-1.00^147^ |

**Supplementary Table 3** Essential information of the oxidation products for the anode

| **Product** | **Half-cell reaction** | $\mathbf{E}^{\mathbf{0}}$ **(V)^*^** | $\boldsymbol{\eta}$ **(V)^**^** | **Market price ($/kg)** |
| --- | --- | --- | --- | --- |
| Oxygen | $\text{2}\text{H}_{\text{2}}\text{O }\text{→}\text{ }\text{O}_{\text{2}}\text{+4}\text{H}^{\text{+}}\text{+4}\text{e}^{\text{-}}$ | 1.23 | 0.37^148,***^  0.25^149^ | 0.024-0.04^150^ |
| Hydrogen peroxide | $\text{2}\text{H}_{\text{2}}\text{O }\text{→}\text{ }\text{H}_{\text{2}}\text{O}_{\text{2}}\text{+2}\text{H}^{\text{+}}\text{+2}\text{e}^{\text{-}}$ | 1.78 | 0.72^151,***^  0.42^152^ | 0.56-0.58^153^ |
| Acetaldehyde | $\text{Ethanol }\text{→}\text{ Acetaldehyde+2}\text{H}^{\text{+}}\text{+2}\text{e}^{\text{-}}$ | 0.193 | 1.26^154^ | 1.00^155^ |
| Acetic acid | $\text{Ethanol+}\text{H}_{\text{2}}\text{O }\text{→}\text{ Acetic acid+4}\text{H}^{\text{+}}\text{+4}\text{e}^{\text{-}}$ | -0.334 | 1.78^156^ | 0.68-0.92^143^ |
| Ethyl acetate | $\text{2Ethanol }\text{→}\text{ Ethyl acetate+4}\text{H}^{\text{+}}\text{+4}\text{e}^{\text{-}}$ | -0.208 | 1.53^157^ | 1.21-1.8^158^ |
| Acrylic acid | $\text{1,3-Propanediol }\text{→}\text{ Acrylic acid+4}\text{H}^{\text{+}}\text{+}\text{4e}^{\text{-}}$ | 0.248 | 0.55^159^ | 2.25-2.88^160^ |
| Lactic acid | $\text{1,2-Propanediol+}\text{H}_{\text{2}}\text{O }\text{→}\text{ Lactic acid+4}\text{H}^{\text{+}}\text{+4}\text{e}^{\text{-}}$ | -0.334 | 0.82^111^ | 1.58-1.87^155^ |
|  | $\text{Glycerol }\text{→}\text{ Lactic acid+2}\text{H}^{\text{+}}\text{+2}\text{e}^{\text{-}}$ | 0.041 | 0.41^161^ |  |
| Benzaldehyde | $\text{Benzyl alcohol}\text{ }\text{→}\text{ B}\text{enzaldehyde+2}\text{H}^{\text{+}}\text{+2}\text{e}^{\text{-}}$ | 0.193 | 1.01^162^ | 1.18-2.11^163^ |
| benzoic acid | $\text{B}\text{enzyl alcohol+}\text{H}_{\text{2}}\text{O}\text{ }\text{→}\text{ }\text{Benzoic acid+4}\text{H}^{\text{+}}\text{+4}\text{e}^{\text{-}}$ | -0.334 | 1.68^33^ | 1.85^164^ |
| 2-Furoic acid | $\text{Furfural+}\text{H}_{\text{2}}\text{O}\text{ }\text{→}\text{ }\text{2-}\text{F}\text{uroic acid+2}\text{H}^{\text{+}}\text{+2}\text{e}^{\text{-}}$ | -1.27 | 2.7^165^ | 6.23^166^ |
|  | $\text{F}\text{urfuryl alcohol+}\text{H}_{\text{2}}\text{O}\text{ }\text{→}\text{ }\text{2-Furoic acid+4}\text{H}^{\text{+}}\text{+4}\text{e}^{\text{-}}$ | -0.515 | 1.88^167^ |  |
| 2,5-Furandicarboxylic acid (FDCA) | $\text{5-}\text{H}\text{ydromethylfurfural (HMF)+}\text{H}_{\text{2}}\text{O}\text{ }\text{→}\text{ }$  $\text{FDCA+6}\text{H}^{\text{+}}\text{+6}\text{e}^{\text{-}}$ | -0.78 | 2.03^119^ | 32 – 580^168^ |
| Acetone | $\text{Isopropanol+}\text{H}_{\text{2}}\text{O}\text{ }\text{→}\text{ A}\text{cetone+2}\text{H}^{\text{+}}\text{+2}\text{e}^{\text{-}}$ | 0.054 | 1.30^169^ | 0.9-1.28^142^ |
| Formaldehyde | $\text{Methanol}\text{ }\text{→}\text{ F}\text{ormaldehyde+2}\text{H}^{\text{+}}\text{+2}\text{e}^{\text{-}}$ | 0.465 | 0.04^170^ | 0.37 - 0.74^171^ |
| formic acid | $\text{M}\text{ethanol+}\text{H}_{\text{2}}\text{O}\text{ }\text{→}\text{ }\text{Formic acid+4}\text{H}^{\text{+}}\text{+4}\text{e}^{\text{-}}$ | -0.258 | 0.76^172^ | 0.97-1.08^132^ |
| Glycolic acid | $\text{Ethylene glycol+}\text{H}_{\text{2}}\text{O}\text{ }\text{→}\text{ G}\text{lycolic acid+4}\text{H}^{\text{+}}\text{+4}\text{e}^{\text{-}}$ | -0.334 | 0.67^173^ | 1.84^174^ |
| Oxalic acid | $\text{E}\text{thylene glycol+2}\text{H}_{\text{2}}\text{O}\text{ }\text{→}\text{ }\text{Oxalic acid+8}\text{H}^{\text{+}}\text{+8}\text{e}^{\text{-}}$ | -0.455 | 0.80 ^175^ | 1.4^176^ |
| ^*^Joback method^177^ was used for unknown free energy, ^**^Based on onset potential, ^***^used in this study | | | | |

**Supplementary Table 4** Base case parameters for technoeconomic analysis

| **Parameters** | **Units** |  |
| --- | --- | --- |
| **Utility cost** | | |
| Steam, 450 psig^71^ | $ kg^-1^ | 0.0145 |
| Steam, 150 psig^71^ | $ kg^-1^ | 0.0105 |
| Steam, 50 psig^71^ | $ kg^-1^ | 0.0066 |
| PV Electricity^178^ | $ kWh^-1^ | 0.1 |
| Process water^71^ | $ m^-3^ | 0.2 |
| KHCO_3_^179^ | $ kg^-1^ | 1.38 |
| Regrigeration, -150^o^F^71^ | $ GJ^-1^ | 12.60 |
| Regrigeration, -90^o^F^71^ | $ GJ^-1^ | 10.30 |
| Regrigeration, -30^o^F^71^ | $ GJ^-1^ | 7.90 |
| Regrigeration, 10^o^F^71^ | $ GJ^-1^ | 5.50 |
| Chilled water, 0^o^F^71^ | $ GJ^-1^ | 4.00 |
| Cooling water^71^ | $ m^-3^ | 0.02 |
| Wastewater treatment^71^ | $ kg^-1^ organics | 0.33 |
| Carbon capture cost^180^ | $ kg^-1^ | 0.06 |
| Direct wages and benefit (DW&B)^71^ | $ operator^-1^ hr^-1^ | 35 |
| Number of workers | - | 10 |
| Tech assistance to manufacturing^71^ | $ shift operator^-1^ yr^-1^ | 60000 |
| Control laboratory^71^ | $ shift operator^-1^ yr^-1^ | 65000 |
| Shift | - | 3 |
| Operating hour | hr yr^-1^ | 8000 |
| **metal market prices** | | |
| Pt^181^ | $ kg^-1^ | 25500 |
| Ir^181^ | $ kg^-1^ | 46940 |
| Al^181^ | $ kg^-1^ | 1.851 |
| Cu^181^ | $ kg^-1^ | 6.04287 |
| Au^181^ | $ kg^-1^ | 41290 |
| Ag^181^ | $ kg^-1^ | 490 |
| Zn^181^ | $ kg^-1^ | 2.57698 |
| Pb^181^ | $ kg^-1^ | 1.97203 |
| Ru^181^ | $ kg^-1^ | 8550 |
| V^182^ | $ kg^-1^ | 175.27 |
| **Economic factors** | | |
| Plant life | Year | 15 |
| Construction period | year | 2 |
| Income tax | % | 38.9 |
| Interest rate | % | 15 |
| MACRS | - | 7-year class |
| **PV Farm** | | |
| Generation capacity | MW | 40 |
| Capacity factor | % | 20 |
| PV efficiency | % | 17 |
| Solar radiation (California) ^183^ | W m^-2^ | 6650 |
| **Operating condition** | | |
| Temperature | oC | 25 |
| Pressure | Pa | 101325 |

**Supplementary Table** **5** Market price of organic raw materials and possible products from the anode side

| **Organic raw materials** | **Market price ($ kg^-1^)** | **Product** |
| --- | --- | --- |
| Ethanol | 0.61-0.75 ^139^ | Acetaldehyde |
|  |  | Acetic acid |
|  |  | Ethyl acetate |
| 1,3-Propanediol | 2.2^184^ | Acrylic acid |
| $\text{1,2-Propanediol}$ $\text{Glycerol}$ | 1.50-2.05^185^  0.97-1.08^186^ | Lactic acid |
| $\text{Benzyl alcohol}$ | 1.92-3.47^187^ | Benzaldehyde |
|  |  | Benzoic acid |
| $\text{Furfural}$  $\text{Furfuryl alcohol}$ | 1.17-1.81^188^  1.25-1.87^189^ | 2-Furoic acid |
| $\text{5-Hydromethylfurfural (HMF)}$ | 1.03^190^ | 2,5-Furandicarboxylic acid (FDCA) |
| $\text{Isopropanol}$ | 1.26-1.6^191^ | Acetone |
| $\text{Methanol}$ | 0.34-0.49 ^134^ | Formaldehyde |
|  |  | Formic acid |
| $\text{Ethylene glycol}$ | 0.83-1.00 ^147^ | Glycolic acid |
|  |  | Oxalic acid |

Supplementary Table 6 Summary of previous technoeconomic analysis for reduction reactions and suggested products which have economic potential via their analysis

| Analyzed chemicals | Year | Economic Metrics | Ref. |
| --- | --- | --- | --- |
| Formic acid | 2011 | Value chain analysis (net present value and payback period) | ^192^ |
| Hydrogen | 2014 | Life cycle net energy assessment using the energy return on energy invested (EROEI) and the energy payback time (EPBT) | ^193^ |
| Methanol | 2015 | The energy incorporation efficiency (EIE) | ^62^ |
| Syngas/Fischer-Tropsch | 2016 | The levelized cost | ^66^ |
| Formic acid | 2016 | Gross margin, benefit/cost ratio (BCR), net present value (NPV) | ^194^ |
| Hydrogen | 2016 | The levelized cost based on NPV | ^130^ |
| Hydrogen, formic acid^*^, carbon monoxide^*^, methanol, methane, ethylene, ethanol | 2016 | Gross margin | ^29^ |
| Carbon monoxide^*^, syngas, formic acid^*^, methanol, methane, ethylene, ethanol, *n*-propanol^*^ | 2018 | NPV | ^131^ |
| Various commodity chemicals (low volume, high-value chemical products such as iodine^*^ and tellurium^*^) | 2018 | Modified Douglas “level 2” infrastructure | ^195^ |
| Carbon monoxide, formic acid, methane, ethylene, propanol^*^, methanol, ethanol, ethylene glycol^*^ | 2018 | The levelized cost based on NPV | ^65^ |
| Diesel fuel^*^, liquid ethanol, formic acid | 2018 | The levelized cost based on NPV | ^196^ |
| ^*^Chemicals that are judged to be economical potential in the study | | | |

**Supplementary Table 7** The lower and upper bound of considered parameters for global sensitivity analysis

| **Parameters** | **Units** | **Base case** | **Lower bound** | **Upper bound** |
| --- | --- | --- | --- | --- |
| Current density | mA cm^-2^ | 500 | 1 | 2000 |
| CO_2_RR FE | % | 90 | 1 | 100 |
| OOR FE | % | 90 | 1 | 100 |
| Cell potential | V vs. RHE | $\text{E}_{\text{anode}}^{\text{0}}\text{+}\text{η}_{\text{anode}}-\text{E}_{\text{cathode}}^{\text{0}}\text{+}\text{η}_{\text{cathode}}$ | **1.** $\text{E}_{\text{anode}}^{\text{0}}\boldsymbol{-}\text{E}_{\text{cathode}}^{\text{0}}\boldsymbol{>0}$  $\text{E}_{\text{anode}}^{\text{0}}-\text{E}_{\text{cathode}}^{\text{0}}+0.01$($\text{η}_{\text{anode}}+\text{η}_{\text{cathode}})$  **2.** $\text{E}_{\text{anode}}^{\text{0}}\boldsymbol{-}\text{E}_{\text{cathode}}^{\text{0}}\boldsymbol{<0}$  $0.01\left( \text{E}_{\text{anode}}^{\text{0}}\text{+}\text{η}_{\text{anode}}-\text{E}_{\text{cathode}}^{\text{0}}\text{+}\text{η}_{\text{cathode}} \right)$ | $\text{E}_{\text{anode}}^{\text{0}}\text{+}\text{η}_{\text{anode}}-\text{E}_{\text{cathode}}^{\text{0}}\text{+}\text{η}_{\text{cathode}}$ |

**Supplementary Table 8** Market analysis and literature survey for the CO2RR and XOR products

| **Product** | **Main applications** | **Commercial production process** | **Economic aspects** |
| --- | --- | --- | --- |
| Hydrogen | Hydrogen is generally used as an important material for ammonia synthesis, refinery processes, coal refinement, the production of synthesis gas, organic and in organic synthesis, and metallurgy in the chemical industry. Except for conventional uses, high temperature during combustion is applied for cutting and autogenous welding in the metal industry by oxyhydrogen flame, and as a heating agent by hydrogen plasma.^197^ | Most of the hydrogen is made from hydrocarbons process including gasification, partial oxidation, and catalytic reforming.  Hydrogen is produced 77% from petrochemicals, 18% from coal, 4 % by electrolysis of aqueous solutions.^198^ | 45-65 Mtonne yr^-1^ for chemical and petrochemical industries, and ~1% of global energy supply.^199^ |
| Carbon monoxide | In commercial petrochemical processes, carbon monoxide is a base material of four principle classes reactions; Reppe chemistry, Koch carbonylation, phosgenation, and ethylene-CO copolymers. These reactions synthesize several chemicals such as acetic acid, acetic anhydride, polycarbonate, formic acid, propionic acid, methyl methacrylate, polyketones and so on. CO is also used as fuel gas, as a reagent for manufacturing special steels or reducing refractory oxides in metallurgy.^200^ | Carbon monoxide is produced as a co-product with hydrogen in the synthetic gas production process. Gas mixtures of CO and H_2_ are separated by pressure swing adsorption or cryogenic distillation.^200^ | 3.757 Mtonne yr^-1^,^201^ |
| Formic acid | Most of formic acid is used as a silage aid and for the preservation of animal biomass because it leaves no residual during evaporation. Other industries also apply its acidity, aldehydic nature, and reducing properties; e.g. a tanning agent in the leather industry, a pH-regulating agent in the textile industry, and a synthetic intermediate for various pharmaceuticals and food chemicals.^202^ | The methyl formate hydrolysis based process produces most of formic acid for the global market. The other production method, formation of free formic acid from formates, occupies approximately ~10% of the installed capacity.^202^ | 0.950 Mtonne yr^-1^ at the end of 2014,^202^ |
| Methanol | Methanol is an important base material for the synthesis of many chemicals; formaldehyde, methyl *tert*-butyl ether, propene, methyl methacrylate, and dimethyl terephthalate. Historically, if prices of petrochemical related to energy use fluctuate, methanol is considered as a fuel for the energy source.^203^ | Catalytic conversion method from synthesis gas achieves a dominant position on an industrial scale process. Its method involves three main processes: production of syngas, synthesis of methanol, and purification of crude methanol.^203^ | Expected over 90 Mtonne yr^-1^,^203^ |
| Methane | A large amount of methane is used exclusively for the production of synthesis gas. Several halogenated chemicals are also produced from methane. Many industries and researchers are developing direct methane conversion to other materials, but they don’t overcome low conversion yields yet.^204^ | The main source of alkane hydrocarbon including methane is natural gas. Methane is condensed from natural gas according to the purification process and used as a fuel and feedstock for syngas.^204^ | 250 Mtonne yr^-1^,^131^ |
| Ethylene | A feed for the production of ethylene oxide, ethylene dichloride and polyethylene accounts for more than 80% of the ethylene consumption. Ethylbenzene, oligomer products, acetaldehyde, and vinyl acetate is also made from ethylene.^205^ | A thermal cracking of petroleum hydrocarbons with steams is a major production source of bulk ethylene. Hot cracked gas of an ethylene furnace is cooled by water quench and compressed; then ethylene is separated by hydrocarbon fractionation section including C_2_H_2_ hydrogenation.^205^ | 112.9 Mtonne yr^-1^ in 2005 ^205^ |
| Ethanol | The main application of ethanol is classified into four categories; a fuel, an alcoholic beverage, a solvent for cosmetics, toiletries, and pharmaceuticals and raw material for several chemicals such as acetaldehyde, butadiene, diethyl ether, ethyl acetate, ethylamine, ethylene, and glycol ethers.^206^ | Ethanol for industrial uses is chemically synthesized by direct or indirect catalytic hydration of ethylene. To make alcoholic beverages, fermentation by yeast or bacteria is generally used for the production of ethanol.^206^ | 58.3 Mtonne yr^-1^ in 2009 ^206^ |
| *n*-Propanol | 1-propanol is the main material of *n-*propyl acetate and used as a solvent in print inks, patin, coatings, and cosmetics. The main application of 2-propanol is a direct solvent and a base material for chemical derivatives including methyl isobutyl ketone, methyl isobutyl carbinol, diisobutyl ketone, and isopropyl acetate.^207^ | The production of 1-propanol has two process steps; firstly, propanal is made by hydroformylation of ethylene, then it is hydrogenated to 1-propanol. Indirect and direct hydration of propene is the major commercial 2-propanol production process.^207^ | 1.4 Mtonne yr^-1^ in 2014 ^207^ |
| Acetaldehyde | Acetaldehyde is used for the production of acetic acid, acetic anhydride, cellulos acetate, vinyl acetate resins, acetate esters, pentaerythritol, synthetic pyridine derivatives, terephtahalic acid, and peracetic acid in the chemical industry. Significant amount of acetladehyde is consumed about the production of perfumes, polyester resins, basic dyes, in fruit and fish preservation. It also used as a favoling agent, a hardeing agent for gelatin and a solvent in the rubber, tanning, and paper industries.^208^ | Depending on the prices of the feedstocks, acetaldehyde is made from oxidation of ethylene, ethyl alcohol, or hydration of acetylene. A rhodium catalyzed process using synthesis gas also produces Acetaldehyde as a coproduct.^208^ | 1.3 Mtonne yr^-1^ produced in 2003 ^209^ |
| Hydroxyacetone | Hydroxyacetone is a base material of hydrogenation for propylene glycol and dehydration for acrolein.  It used to add flavor in the food industry and as a reducing agent in the textile industry.^210^ | Hydroxyacetone is a by-product of the cumene-phenol process, but it is considered as an impurity.^211^ It is also an intermediate product on glycerol hydrogenation.^212^ Propargyl alcohol reacts to waters with acidic mercuric salt catalysts to give hydroxyacetone.^213^ | - |
| Acetone | Acetone is mainly used as a direct solvent in various applications and for the production of methyl methacrylate, bisphenol A, and aldol chemicals.^214^ | Cumene process which occupies more than 90% of acetone production process consists of two steps; firstly, Friedel-Crafts alkylation produces cumene from benzene and propene, then cumene oxidation process, named Hock process, produces acetone from oxidized cumene, cumene hydroperoxide.^214^ | More than 6 Mtonne yr^-1^, 6.4 Mtonne yr^-1^ in 2007 ^214^ |
| Acetic acid | Most of acetic acid use is for the production of vinyl acetate, terephthalic acid, and acetate esters in the chemical industry. Smaller uses are for the manufacture of acetic anhydride, chloroacetic acid, and keten derivatives.^215^ | Synthetic acetic acid is produced by methanol carbonylation, acetaldehyde oxidation, butane and naphtha oxidation, and methyl acetate carbonylation.^215^ | 10.2 Mtonne yr^-1^ in 2011, expected to reach 15.5 Mtonne yr^-1^ by 2020 ^215^ |
| Allyl alcohol | The conventional uses of allyl alcohol were classified to two applications; as a base material for glycerol, and a monomer, diethylene glycol bis(allyl carbonate), for the plastic optical lens. Most of ally alcohol is currently used for the production of 1,4-butanediol. In addition to that, allyl ether, allyl esters, allyl glycol, allyl ester oligomers, and allyl-styrene copolymers are produced by allyl alcohol.^216^ | The major two routes are available to produce allyl alcohol on an industrial scale at present. One is isomerization of propylene oxide. The second process includes the acetoxylation of propylene and the hydrolysis of allyl acetate with an acid catalyst.^216^ | 0.136 Mtonne yr^-1^ in 2005 ^217^  0.280 Mtonne yr^-1^ ^216^ |
| Glycolaldehyde | The main application of glycolaldehyde is a food browning agent. It also reacts as an intermediate in the production of ethylene glycol or ethanolamine from glucose or cellulose.^218^ It is a base material for the synthesis of serine, tryptophan, medicines and agrochemical.^219^ | Dehydrogenation of ethylene glycol with the metal catalyst is a conventional chemical method for the production of glycolaldehyde. At present, it is a major co-product of biorefinery which produces pyrolysis oil.^218^ | - |
| Propionaldehyde | Propionaldehyde is widely used as an intermediate in the chemical industry, especially reduction, oxidation, and condensation. Major applications of propionaldehyde are hydrogenation for n-propanol and oxidation for propionic acid. Smaller amounts are used for condensation to trimethylolethane.^220^ | A major route for the propionaldehyde production is oxo synthesis which adds a double bond olefin to H_2_ and CO with a transition-metal catalyst.^220^ | 0.466 Mtonne yr^-1^ in 2014 ^220^ |
| Ethylene glycol | Ethylene glycol is an important raw material for the production of plastics; e.g., Poly(ethylene terephthalate) bottle-grade resins, and polyesters fibers. It is also used as an antifreeze and a deicing agent in various industries, especially liquid-cooled moto vehicles.^221^ | A dominant process for ethylene glycol is based on the ethylene oxide hydrolysis at present. Production from formaldehyde and CO, and direct oxidation of ethylene were considered as alternative methods in commercial scale, but abandoned because of their drawbacks.^221^ | 20 Mtonne yr^-1^ in 2010 ^222^ |
| Oxygen | Large amounts of oxygen are used for the combustion and metallurgy, especially steel-making. In the chemical industry, commercial oxygen as a raw material of synthesis reaction, via partial oxidation, has a trade-off compared to air. Other applications of oxygen are oxygen bleaching of wood pulp, wastewater treatment, and medical application.^223^ | High purity oxygen, more than 95% purity, is produced by cryogenic air separation. In the case of low purity, 90-93%, pressure swing adsorption is considered to the major route for oxygen production. At present, membrane techniques are also employed in air separation processes.^223^ | Close to 2 Mtonne day^-1^ ^223^ |
| Hydrogen peroxide | Hydrogen peroxide is mainly used for bleaching fibers in the textile industry. It is a raw material for the production of peroxy compounds, epoxidation, and hydroxylation in the chemical industry. Cleaning silicon wafers and removing photoresist layers in wet chemical process, and detoxifying effluents containing formaldehyde, phenols or cyanide are other industrial applications.^224^ | Major processes for hydrogen peroxide are Organic Autoxidation Processes including Anthraquinone Process (AO Process) and 2-Propanol Process (Shell Process)^224^ | > 3 Mtonne yr^-1^,^225^ |
| Ethyl acetate | Ethyl acetate is a solvent for coatings, adhesives, ink, cosmetics, film base, synthesis in the pharmaceutical industry, and for the decaffeination of coffee in an extraction process.^226^ | Ethyl acetate is produced from ethanol and acetic acid in batch or continuous process including the distillation for the removal of the ester, or the Tishchenko reaction using acetaldehyde.^226^ | Global capacity : 2.7 Mtonne yr^-1^,^227^ |
| Acrylic acid | Acrylic acid is an intermediate in the production of acrylates. Poly(Acrylic acids) are used as flocculants and dispersants with the polymeric sodium salts in the chemical industry.^228^ | Major acrylic acid production process is a heterogeneous catalytic oxidation process of Propene including separation process for the water and solvent removal.^228^ | >3.4 Mtonne yr^-1^ in 2002 ^228^ |
| Lactic acid | Lactic acid is an acidulant and preservative in the food industry and a monomer of polylactic acid or polylactide (PLA) in the polymer industry. It also used as animal feed in agricultural fields. A variety of industrial uses including metal plating, cosmetics, textile and leather industry, the manufacture of paints, electronics, and metal cleaning.^229^ | Lactic acid is produced by the fermentation process or a synthetic method for an industrial scale.^229^ | 0.45 Mtonne yr^-1^,^229^ |
| Benzaldehyde | Benzaldehyde is considered as a base material for the production of odorants and favor in the perfume and flavor industries. It is also used for the production of triphenylmethane dyes and other chemical intermediates, such as benzoin, benzylamine, benzyl alcohol, mandelic acid, and benzylideneacetone. In other industries, it is used as a corrosion inhibitor for photochemistry, dyeing auxiliary for the electroplating industry.^230^ | Major routes of the benzaldehyde production are the hydrolysis of benzal chloride or the partial oxidation of toluene.^230^ | - |
| Benzoic acid | Benzoic acid is mainly used as a raw material in the production of phenol, caprolactam, glycol dibenzoate esters, and sodium benzoate.^231^ | Benzoic acid is produced by liquid phase toluene oxidation with cobalt salts.^231^ | The capacity of the North American producers is estimated 0.130 Mtonne yr^-1^, but they consume most of them.^231^ |
| 2-Furoic acid | Furoic acid is considered as a base material for the production of pharmaceutical, agricultural and industrial chemicals. Significant amounts are used for the conversion of 2-furoyl chloride.^232^ | Cannizzaro reaction produces furoic acid salts and furfuryl alcohol in equimolar. Filtration and acidification process after reaction purify these salts sublimated to crude furoic acid. Oxidation of furfural with oxygen or hydrogen peroxide was considered to another method for furoic acid, but its application is not employed in large scales.^233^ | - |
| 2,5-Furandicarboxylic acid (FDCA) | FDCA is expected to replace conventional petrochemical intermediates used for the synthesis of plastics, especially polyethylene terephthalate (PET).^234^ | Reaction routes for the synthesis of FDCA is classified into three methods; the dehydration of hexose derivatives, the oxidation of 2,5-disubstituted furans, and catalytic conversions of various furan derivatives.^234^ | Estimated production is 3.5-5 tonne yr^-1^ in 2012 and 0.5 Mtonne yr^-1^ in 2018,^234^ |
| Formaldehyde | Formaldehyde is used for the production of resins and as an intermediate for synthesizing a variety of chemical compounds, such as 1,4-butanediol, trimethylolpropane, and neopentyl glycol.^235^ | There are two formaldehyde production processes; Silver catalyst process including partial oxidation and dehydrogenation with air, steam and excess methanol (BASF process), and Formox process with a metal oxide catalyst for oxidation of excess air and methanol.^235^ | 18 Mtonne yr^-1^ in 2011^235^ |
| Glycolic acid | Glycolic acid is used in textile dyeing, printing, and crease proofing. It also is an incorporation chemical into acidic cleansing agents, especially cleansing operations for milk-processing equipment, or a rust and scale removal agent in heat exchangers and pipelines.^236^ | Commercial glycolic acid process is classified to treatments of formaldehyde or trioxymethylene with carbon monoxide and water in acid catalyst at 30 MPa (Du Pont), and electrolytic reduction of oxalic acid (Degussa).^236^ | 2000-3000 tonne yr^-1^,^236^ |
| Oxalic acid | For metal treatment, oxalic acid salt is used for various purposes, such as rust removal, removal of iron veins in marbles, metal cleansers with rust protection, anodizing of aluminum, the formation of very hard, abrasion and corrosion protective coatings. In chemical industries, it used for preparation of esters and salts, the reagent in chemical synthesis, and concertation of rare earth elements. Other applications are the printing and dyeing of wool and cotton, the tanning and bleaching of leather, and bleaching of cork, wood, or natural and synthetic waxes.^237^ | There are two routes for manufacturing oxalic acid; oxidation of carbohydrates, ethylene glycol or propene, and synthesis process including the reaction of carbon monoxide and lower alcohol, and hydrolyzation of diester which is reaction products.^237^ | 0.190 Mtonne during 2007-2009 ^237^ |

**Supplementary Table 9** Overall assessment of electrochemical coproduction technology in **base case**, **optimal case I** (CD: 2000 mA cm^-2^_­_, FE: 99%, η: 50%, electricity cost: 0.06 $ kWh^-1^, raw material cost (CO_2_): $0 kg^‑1­^_­_, and no tax), and **optimal case II** (CD: 2000 mA cm^-2^_­_, FE: 99%, η: 10%, electricity cost (Levelized cost of energy (LCOE) of subsidized solar PV in 2018): 0.032 $ kWh^-1^,^238^ raw material cost (CO_2_): $0 kg^‑1­^_­_, no tax, electrolyzer cost of future central case,^239^ lower cost of organic raw materials, and recycle ratio: 95%). The levelized cost is lower than the market price at base case (blue), optimal case I (green), optimal case II (purple), optimal case II with 20% margin (red)

| **Name** | | **Base case** | | **Optimal I** | | **Optimal II** | |
| --- | --- | --- | --- | --- | --- | --- | --- |
| **Cathode** | **Anode** | **NPV ($)** | **LC/MC** | **NPV ($)** | **LC/MC** | **NPV ($)** | **LC/MC** |
| **Ethylene** | **2,5-Furandicarboxylic acid (FDCA)** | 2.05E+10 | 9.00E-03 | 7.50E+10 | 6.46E-03 | 3.78E+11 | 4.18E-03 |
| **Ethylene glycol** | **2,5-Furandicarboxylic acid (FDCA)** | 2.01E+10 | 9.95E-03 | 7.33E+10 | 7.10E-03 | 3.70E+11 | 4.23E-03 |
| ***n-*Propanol** | **2,5-Furandicarboxylic acid (FDCA)** | 1.85E+10 | 9.24E-03 | 6.74E+10 | 6.56E-03 | 3.40E+11 | 4.77E-03 |
| **Methanol** | **2,5-Furandicarboxylic acid (FDCA)** | 1.78E+10 | 9.15E-03 | 6.51E+10 | 6.52E-03 | 3.28E+11 | 4.21E-03 |
| **Ethanol** | **2,5-Furandicarboxylic acid (FDCA)** | 1.74E+10 | 9.47E-03 | 6.36E+10 | 6.64E-03 | 3.20E+11 | 4.23E-03 |
| **Propionaldehyde** | **2,5-Furandicarboxylic acid (FDCA)** | 1.63E+10 | 9.82E-03 | 5.96E+10 | 6.96E-03 | 3.01E+11 | 4.16E-03 |
| **Allyl alcohol** | **2,5-Furandicarboxylic acid (FDCA)** | 1.63E+10 | 9.80E-03 | 5.94E+10 | 6.95E-03 | 3.00E+11 | 3.12E-03 |
| **Carbon monoxide** | **2,5-Furandicarboxylic acid (FDCA)** | 1.62E+10 | 1.48E-02 | 5.94E+10 | 1.16E-02 | 3.01E+11 | 3.38E-03 |
| **Hydroxyacetone (acetol)** | **2,5-Furandicarboxylic acid (FDCA)** | 1.62E+10 | 1.03E-02 | 5.93E+10 | 7.29E-03 | 3.00E+11 | 3.15E-03 |
| **Formic acid** | **2,5-Furandicarboxylic acid (FDCA)** | 1.61E+10 | 1.50E-02 | 5.87E+10 | 1.14E-02 | 2.97E+11 | 6.69E-03 |
| **Acetaldehyde** | **2,5-Furandicarboxylic acid (FDCA)** | 1.60E+10 | 9.75E-03 | 5.86E+10 | 6.96E-03 | 2.95E+11 | 4.39E-03 |
| **Acetone** | **2,5-Furandicarboxylic acid (FDCA)** | 1.60E+10 | 9.64E-03 | 5.83E+10 | 6.79E-03 | 2.94E+11 | 3.51E-03 |
| **Acetic acid** | **2,5-Furandicarboxylic acid (FDCA)** | 1.60E+10 | 1.09E-02 | 5.83E+10 | 7.89E-03 | 2.94E+11 | 4.53E-03 |
| **Glycolaldehyde** | **2,5-Furandicarboxylic acid (FDCA)** | 1.54E+10 | 1.10E-02 | 5.63E+10 | 7.83E-03 | 2.84E+11 | 4.54E-03 |
| **Methane** | **2,5-Furandicarboxylic acid (FDCA)** | 1.52E+10 | 9.04E-03 | 5.55E+10 | 6.11E-03 | 2.79E+11 | 3.96E-03 |
| **Ethylene** | **2-Furoic acid (from Furfural)** | 5.09E+08 | 3.79E-01 | 2.05E+09 | 3.18E-01 | 1.22E+10 | 1.98E-01 |
| **Ethylene glycol** | **2-Furoic acid (from Furfural)** | 4.94E+08 | 3.94E-01 | 2.01E+09 | 3.27E-01 | 1.21E+10 | 1.97E-01 |
| ***n-*Propanol** | **2-Furoic acid (from Furfural)** | 4.63E+08 | 3.83E-01 | 1.89E+09 | 3.17E-01 | 1.08E+10 | 2.22E-01 |
| **Methanol** | **2-Furoic acid (from Furfural)** | 4.48E+08 | 3.80E-01 | 1.81E+09 | 3.18E-01 | 1.04E+10 | 2.23E-01 |
| **Ethanol** | **2-Furoic acid (from Furfural)** | 4.35E+08 | 3.86E-01 | 1.77E+09 | 3.21E-01 | 1.02E+10 | 2.19E-01 |
| **Allyl alcohol** | **2-Furoic acid (from Furfural)** | 4.12E+08 | 3.91E-01 | 1.68E+09 | 3.24E-01 | 9.71E+09 | 2.23E-01 |
| **Propionaldehyde** | **2-Furoic acid (from Furfural)** | 4.02E+08 | 3.98E-01 | 1.64E+09 | 3.29E-01 | 9.52E+09 | 2.27E-01 |
| **Hydroxyacetone (acetol)** | **2-Furoic acid (from Furfural)** | 3.98E+08 | 4.05E-01 | 1.63E+09 | 3.34E-01 | 9.58E+09 | 2.24E-01 |
| **Acetone** | **2-Furoic acid (from Furfural)** | 4.02E+08 | 3.89E-01 | 1.63E+09 | 3.24E-01 | 9.48E+09 | 2.19E-01 |
| **Acetaldehyde** | **2-Furoic acid (from Furfural)** | 4.01E+08 | 3.93E-01 | 1.63E+09 | 3.28E-01 | 9.44E+09 | 2.26E-01 |
| **Acetic acid** | **2-Furoic acid (from Furfural)** | 3.87E+08 | 4.14E-01 | 1.58E+09 | 3.47E-01 | 9.30E+09 | 2.36E-01 |
| **Methane** | **2-Furoic acid (from Furfural)** | 3.83E+08 | 3.82E-01 | 1.57E+09 | 3.12E-01 | 8.99E+09 | 2.15E-01 |
| **Glycolaldehyde** | **2-Furoic acid (from Furfural)** | 3.79E+08 | 4.13E-01 | 1.55E+09 | 3.45E-01 | 9.40E+09 | 2.09E-01 |
| **Formic acid** | **2-Furoic acid (from Furfural)** | 3.64E+08 | 4.80E-01 | 1.52E+09 | 4.09E-01 | 9.48E+09 | 2.64E-01 |
| **Carbon monoxide** | **2-Furoic acid (from Furfural)** | 3.39E+08 | 5.04E-01 | 1.43E+09 | 4.28E-01 | 9.13E+09 | 2.65E-01 |
| **Ethylene** | **2-Furoic acid (from Furfuryl alcohol)** | 2.03E+08 | 5.25E-01 | 9.34E+08 | 4.04E-01 | 5.74E+09 | 2.71E-01 |
| **Ethylene glycol** | **2-Furoic acid (from Furfuryl alcohol)** | 1.94E+08 | 5.49E-01 | 9.09E+08 | 4.23E-01 | 5.81E+09 | 2.67E-01 |
| ***n-*Propanol** | **2-Furoic acid (from Furfuryl alcohol)** | 1.86E+08 | 5.25E-01 | 8.61E+08 | 4.05E-01 | 5.33E+09 | 2.67E-01 |
| **Methanol** | **2-Furoic acid (from Furfuryl alcohol)** | 1.79E+08 | 5.24E-01 | 8.21E+08 | 4.05E-01 | 5.06E+09 | 2.71E-01 |
| **Ethanol** | **2-Furoic acid (from Furfuryl alcohol)** | 1.72E+08 | 5.32E-01 | 7.98E+08 | 4.09E-01 | 4.94E+09 | 2.72E-01 |
| **Allyl alcohol** | **2-Furoic acid (from Furfuryl alcohol)** | 1.65E+08 | 5.39E-01 | 7.72E+08 | 4.11E-01 | 4.82E+09 | 2.68E-01 |
| **Acetone** | **2-Furoic acid (from Furfuryl alcohol)** | 1.58E+08 | 5.38E-01 | 7.32E+08 | 4.17E-01 | 4.59E+09 | 2.73E-01 |
| **Acetaldehyde** | **2-Furoic acid (from Furfuryl alcohol)** | 1.56E+08 | 5.45E-01 | 7.31E+08 | 4.22E-01 | 4.61E+09 | 2.76E-01 |
| **Propionaldehyde** | **2-Furoic acid (from Furfuryl alcohol)** | 1.53E+08 | 5.58E-01 | 7.26E+08 | 4.28E-01 | 4.61E+09 | 2.78E-01 |
| **Hydroxyacetone (acetol)** | **2-Furoic acid (from Furfuryl alcohol)** | 1.50E+08 | 5.69E-01 | 7.19E+08 | 4.38E-01 | 4.60E+09 | 2.84E-01 |
| **Methane** | **2-Furoic acid (from Furfuryl alcohol)** | 1.51E+08 | 5.24E-01 | 7.13E+08 | 3.92E-01 | 4.39E+09 | 2.55E-01 |
| **Acetic acid** | **2-Furoic acid (from Furfuryl alcohol)** | 1.42E+08 | 5.87E-01 | 6.85E+08 | 4.58E-01 | 4.57E+09 | 2.81E-01 |
| **Glycolaldehyde** | **2-Furoic acid (from Furfuryl alcohol)** | 1.42E+08 | 5.81E-01 | 6.77E+08 | 4.55E-01 | 4.42E+09 | 2.92E-01 |
| **Formic acid** | **2-Furoic acid (from Furfuryl alcohol)** | 1.17E+08 | 6.92E-01 | 6.07E+08 | 5.66E-01 | 4.63E+09 | 3.41E-01 |
| **Carbon monoxide** | **2-Furoic acid (from Furfuryl alcohol)** | 8.43E+07 | 7.66E-01 | 4.97E+08 | 6.22E-01 | 4.02E+09 | 3.79E-01 |
| **Allyl alcohol** | **Lactic acid (from glycerol)** | -5.63E+07 | 1.21E+00 | 1.95E+07 | 9.81E-01 | 1.22E+09 | 7.59E-01 |
| ***n-*Propanol** | **Lactic acid (from glycerol)** | -6.86E+07 | 1.21E+00 | 1.81E+07 | 9.85E-01 | 1.68E+09 | 7.14E-01 |
| **Methane** | **Lactic acid (from glycerol)** | -5.69E+07 | 1.25E+00 | 2.02E+06 | 9.98E-01 | 8.25E+08 | 7.99E-01 |
| **Ethylene glycol** | **Lactic acid (from glycerol)** | -9.33E+07 | 1.25E+00 | -3.42E+07 | 1.02E+00 | 1.42E+09 | 7.94E-01 |
| **Ethylene** | **Lactic acid (from glycerol)** | -8.90E+07 | 1.24E+00 | -2.52E+07 | 1.02E+00 | 1.37E+09 | 7.97E-01 |
| ***n-*Propanol** | **Glycolic acid** | -7.97E+07 | 1.47E+00 | -2.28E+07 | 1.04E+00 | 1.03E+09 | 6.69E-01 |
| **Ethanol** | **Lactic acid (from glycerol)** | -7.39E+07 | 1.26E+00 | -1.98E+07 | 1.02E+00 | 1.01E+09 | 8.05E-01 |
| **Allyl alcohol** | **Glycolic acid** | -6.56E+07 | 1.44E+00 | -9.11E+06 | 1.02E+00 | 1.00E+09 | 6.36E-01 |
| **Methanol** | **Lactic acid (from glycerol)** | -6.88E+07 | 1.23E+00 | -1.50E+07 | 1.01E+00 | 9.66E+08 | 8.07E-01 |
| **Acetaldehyde** | **Lactic acid (from glycerol)** | -6.80E+07 | 1.27E+00 | -2.61E+07 | 1.03E+00 | 9.37E+08 | 8.01E-01 |
| **Ethylene** | **Glycolic acid** | -1.06E+08 | 1.55E+00 | -8.68E+07 | 1.12E+00 | 9.21E+08 | 7.34E-01 |
| **Formic acid** | **Lactic acid (from glycerol)** | -1.37E+08 | 1.44E+00 | -2.29E+08 | 1.21E+00 | 8.79E+08 | 7.91E-01 |
| **Hydroxyacetone (acetol)** | **Lactic acid (from glycerol)** | -7.95E+07 | 1.30E+00 | -6.73E+07 | 1.07E+00 | 8.47E+08 | 8.23E-01 |
| **Propionaldehyde** | **Lactic acid (from glycerol)** | -7.85E+07 | 1.31E+00 | -5.72E+07 | 1.06E+00 | 8.39E+08 | 8.21E-01 |
| **Glycolaldehyde** | **Glycolic acid** | -8.66E+07 | 1.64E+00 | -9.16E+07 | 1.18E+00 | 8.12E+08 | 6.74E-01 |
| **Methanol** | **Glycolic acid** | -8.43E+07 | 1.56E+00 | -6.00E+07 | 1.11E+00 | 8.00E+08 | 7.09E-01 |
| **Ethanol** | **Glycolic acid** | -8.37E+07 | 1.58E+00 | -6.13E+07 | 1.12E+00 | 7.54E+08 | 7.16E-01 |
| **Acetone** | **Glycolic acid** | -7.32E+07 | 1.56E+00 | -5.32E+07 | 1.11E+00 | 7.09E+08 | 7.03E-01 |
| **Acetaldehyde** | **Glycolic acid** | -7.78E+07 | 1.59E+00 | -6.80E+07 | 1.14E+00 | 7.02E+08 | 7.12E-01 |
| **Formic acid** | **Glycolic acid** | -1.47E+08 | 1.76E+00 | -2.93E+08 | 1.41E+00 | 6.77E+08 | 8.11E-01 |
| **Allyl alcohol** | **Ethyl acetate** | -6.50E+07 | 1.87E+00 | -7.61E+07 | 1.28E+00 | 6.57E+08 | 5.24E-01 |
| **Allyl alcohol** | **Lactic acid (from 1,2-propandiol)** | -1.25E+08 | 1.85E+00 | -2.27E+08 | 1.42E+00 | 6.33E+08 | 7.67E-01 |
| **Hydroxyacetone (acetol)** | **Glycolic acid** | -9.18E+07 | 1.68E+00 | -9.91E+07 | 1.20E+00 | 6.30E+08 | 7.45E-01 |
| **Propionaldehyde** | **Glycolic acid** | -8.87E+07 | 1.69E+00 | -8.78E+07 | 1.19E+00 | 6.07E+08 | 7.43E-01 |
| **Methane** | **Glycolic acid** | -6.31E+07 | 1.58E+00 | -2.50E+07 | 1.06E+00 | 6.28E+08 | 6.86E-01 |
| **Glycolaldehyde** | **Lactic acid (from glycerol)** | -7.93E+07 | 1.31E+00 | -6.38E+07 | 1.07E+00 | 6.06E+08 | 8.16E-01 |
| **Acetic acid** | **Glycolic acid** | -1.02E+08 | 1.76E+00 | -1.32E+08 | 1.27E+00 | 5.32E+08 | 7.84E-01 |
| **Hydroxyacetone (acetol)** | **Ethyl acetate** | -8.05E+07 | 2.21E+00 | -1.34E+08 | 1.55E+00 | 3.42E+08 | 7.19E-01 |
| **Acetone** | **Lactic acid (from glycerol)** | -6.36E+07 | 1.25E+00 | -2.08E+07 | 1.03E+00 | 2.90E+08 | 8.70E-01 |
| **Ethylene glycol** | **Ethyl acetate** | -8.80E+07 | 2.03E+00 | -1.29E+08 | 1.41E+00 | 2.83E+08 | 8.18E-01 |
| **Allyl alcohol** | **Oxalic acid** | -9.42E+07 | 2.13E+00 | -1.38E+08 | 1.45E+00 | 2.80E+08 | 8.18E-01 |
| **Glycolaldehyde** | **Acetic acid** | -9.15E+07 | 3.54E+00 | -1.73E+08 | 2.32E+00 | 2.71E+08 | 5.89E-01 |
| **Acetic acid** | **Lactic acid (from glycerol)** | -8.84E+07 | 1.34E+00 | -9.08E+07 | 1.12E+00 | 2.46E+08 | 8.54E-01 |
| **Glycolaldehyde** | **Ethyl acetate** | -7.92E+07 | 2.12E+00 | -1.36E+08 | 1.53E+00 | 2.11E+08 | 8.37E-01 |
| **Allyl alcohol** | **Acrylic acid** | -1.13E+08 | 1.80E+00 | -2.10E+08 | 1.44E+00 | 1.67E+08 | 8.91E-01 |
| ***n-*Propanol** | **Acetaldehyde** | -8.97E+07 | 2.67E+00 | -1.53E+08 | 1.79E+00 | 1.55E+08 | 7.88E-01 |
| **Glycolaldehyde** | **Oxalic acid** | -1.13E+08 | 2.50E+00 | -2.10E+08 | 1.77E+00 | 1.49E+08 | 8.91E-01 |
| **Hydroxyacetone (acetol)** | **Lactic acid (from 1,2-propandiol)** | -1.50E+08 | 2.12E+00 | -3.23E+08 | 1.66E+00 | 1.42E+08 | 9.42E-01 |
| **Acetaldehyde** | **Ethyl acetate** | -7.04E+07 | 2.07E+00 | -1.07E+08 | 1.44E+00 | 1.37E+08 | 8.87E-01 |
| **Acetone** | **Ethyl acetate** | -6.92E+07 | 2.07E+00 | -1.07E+08 | 1.45E+00 | 1.36E+08 | 8.86E-01 |
| ***n-*Propanol** | **Oxalic acid** | -1.15E+08 | 2.34E+00 | -1.84E+08 | 1.58E+00 | 1.32E+08 | 9.18E-01 |
| **Formic acid** | **Ethyl acetate** | -1.13E+08 | 2.13E+00 | -2.42E+08 | 1.65E+00 | 1.27E+08 | 9.32E-01 |
| ***n-*Propanol** | **Formic acid** | -1.10E+08 | 2.98E+00 | -1.69E+08 | 1.76E+00 | 1.23E+08 | 8.89E-01 |
| **Ethanol (Cascade)** | **Ethyl acetate (Cascade)** | -7.46E+07 | 2.19E+00 | -1.10E+08 | 1.48E+00 | 1.16E+08 | 8.18E-01 |
| **Ethanol** | **Ethyl acetate** | -7.54E+07 | 2.12E+00 | -1.13E+08 | 1.46E+00 | 1.13E+08 | 9.08E-01 |
| **Formic acid** | **Lactic acid (from 1,2-propandiol)** | -2.07E+08 | 2.11E+00 | -4.85E+08 | 1.71E+00 | 1.02E+08 | 9.70E-01 |
| **Methane** | **Ethyl acetate** | -6.27E+07 | 2.14E+00 | -8.27E+07 | 1.41E+00 | 1.08E+08 | 8.94E-01 |
| **Ethanol** | **Acrylic acid** | -1.30E+08 | 1.95E+00 | -2.54E+08 | 1.56E+00 | 9.40E+07 | 9.30E-01 |
| **Allyl alcohol** | **Formaldehyde** | -1.33E+08 | 5.08E+00 | -2.02E+08 | 2.50E+00 | 6.77E+07 | 7.48E-01 |
| **Ethylene glycol** | **Formic acid** | -1.42E+08 | 2.99E+00 | -2.38E+08 | 1.88E+00 | 6.66E+07 | 9.51E-01 |
| **Ethylene glycol (Cascade)** | **Glycolic acid (Cascade)** | -1.08E+08 | 1.60E+00 | -6.31E+07 | 1.10E+00 | 5.69E+07 | 9.85E-01 |
| ***n-*Propanol** | **Ethyl acetate** | -7.23E+07 | 1.96E+00 | -9.82E+07 | 1.35E+00 | 3.46E+07 | 9.75E-01 |
| **Ethylene glycol** | **Glycolic acid** | -1.13E+08 | 1.55E+00 | -7.86E+07 | 1.10E+00 | 2.89E+07 | 9.92E-01 |
| **Hydroxyacetone (acetol)** | **Hydrogen Peroxide** | -6.22E+07 | 4.37E+00 | -8.55E+07 | 2.75E+00 | 2.74E+07 | 6.44E-01 |
| **Carbon monoxide** | **Ethyl acetate** | -1.44E+08 | 2.93E+00 | -3.67E+08 | 2.34E+00 | 8.44E+07 | 9.32E-01 |
| **Methane** | **Formic acid** | -8.88E+07 | 4.02E+00 | -1.35E+08 | 2.12E+00 | 3.81E+07 | 9.37E-01 |
| **Acetone** | **Formic acid** | -1.00E+08 | 3.26E+00 | -1.61E+08 | 1.94E+00 | 1.69E+07 | 9.80E-01 |
| **Formic acid** | **Formic acid** | -1.66E+08 | 2.65E+00 | -3.68E+08 | 1.97E+00 | 1.50E+07 | 9.92E-01 |
| **Acetaldehyde** | **Formic acid** | -1.03E+08 | 3.26E+00 | -1.78E+08 | 2.00E+00 | 1.44E+07 | 9.84E-01 |
| **Propionaldehyde** | **Hydrogen Peroxide** | -6.14E+07 | 4.93E+00 | -7.90E+07 | 3.02E+00 | 8.71E+06 | 8.42E-01 |
| **Acetone** | **Oxalic acid** | -1.01E+08 | 2.53E+00 | -1.79E+08 | 1.74E+00 | 8.46E+06 | 9.93E-01 |
| **Glycolaldehyde** | **Oxygen** | -8.83E+07 | 7.47E+00 | -1.28E+08 | 4.77E+00 | 7.39E+06 | 8.47E-01 |
| **Acetaldehyde** | **Oxalic acid** | -1.06E+08 | 2.58E+00 | -1.94E+08 | 1.78E+00 | -3.34E+06 | 1.00E+00 |
| **Glycolaldehyde** | **Formic acid** | -1.12E+08 | 3.05E+00 | -2.00E+08 | 1.98E+00 | -3.18E+06 | 1.00E+00 |
| **Formic acid** | **Oxalic acid** | -1.75E+08 | 2.38E+00 | -4.20E+08 | 1.88E+00 | -3.39E+07 | 1.01E+00 |
| **Allyl alcohol** | **Acetaldehyde** | -8.02E+07 | 2.43E+00 | -1.33E+08 | 1.67E+00 | -7.77E+06 | 1.01E+00 |
| **Acetic acid** | **Formic acid** | -1.23E+08 | 3.52E+00 | -2.39E+08 | 2.31E+00 | -9.97E+06 | 1.01E+00 |
| **Allyl alcohol** | **Formic acid** | -9.13E+07 | 2.54E+00 | -1.20E+08 | 1.53E+00 | -2.72E+07 | 1.02E+00 |
| **Ethanol** | **Formic acid** | -1.11E+08 | 3.48E+00 | -1.79E+08 | 2.03E+00 | -1.92E+07 | 1.02E+00 |
| **Ethylene glycol** | **Acrylic acid** | -1.62E+08 | 1.93E+00 | -3.25E+08 | 1.53E+00 | -7.94E+07 | 1.03E+00 |
| **Methanol** | **Oxalic acid** | -1.18E+08 | 2.65E+00 | -2.12E+08 | 1.81E+00 | -4.68E+07 | 1.04E+00 |
| ***n-*Propanol** | **Lactic acid (from 1,2-propandiol)** | -1.51E+08 | 1.92E+00 | -2.92E+08 | 1.49E+00 | -1.23E+08 | 1.04E+00 |
| **Allyl alcohol** | **Acetic acid** | -7.79E+07 | 3.02E+00 | -1.26E+08 | 1.89E+00 | -2.48E+07 | 1.03E+00 |
| **Methane** | **Oxalic acid** | -8.88E+07 | 2.78E+00 | -1.40E+08 | 1.77E+00 | -1.68E+07 | 1.02E+00 |
| **Ethanol** | **Oxalic acid** | -1.16E+08 | 2.65E+00 | -2.07E+08 | 1.81E+00 | -5.80E+07 | 1.05E+00 |
| **Methanol** | **Formic acid** | -1.11E+08 | 3.47E+00 | -1.83E+08 | 2.05E+00 | -4.32E+07 | 1.05E+00 |
| **Ethylene glycol** | **Lactic acid (from 1,2-propandiol)** | -1.91E+08 | 1.98E+00 | -3.79E+08 | 1.53E+00 | -2.06E+08 | 1.06E+00 |
| **Acetaldehyde** | **Acrylic acid** | -1.24E+08 | 1.97E+00 | -2.33E+08 | 1.55E+00 | -6.89E+07 | 1.06E+00 |
| ***n-*Propanol** | **Acrylic acid** | -1.32E+08 | 1.87E+00 | -2.43E+08 | 1.48E+00 | -1.02E+08 | 1.07E+00 |
| **Acetone** | **Lactic acid (from 1,2-propandiol)** | -1.33E+08 | 2.03E+00 | -2.66E+08 | 1.56E+00 | -1.70E+08 | 1.07E+00 |
| **Glycolaldehyde** | **Lactic acid (from 1,2-propandiol)** | -1.45E+08 | 2.07E+00 | -2.99E+08 | 1.61E+00 | -1.81E+08 | 1.07E+00 |
| **Hydroxyacetone (acetol)** | **Oxalic acid** | -1.20E+08 | 2.75E+00 | -2.28E+08 | 1.91E+00 | -9.11E+07 | 1.07E+00 |
| **Acetaldehyde** | **Lactic acid (from 1,2-propandiol)** | -1.36E+08 | 2.04E+00 | -2.75E+08 | 1.57E+00 | -1.85E+08 | 1.08E+00 |
| **Methanol (Cascade)** | **Formic acid (Cascade)** | -1.10E+08 | 3.84E+00 | -1.81E+08 | 2.20E+00 | -5.57E+07 | 1.08E+00 |
| **Acetic acid** | **Lactic acid (from 1,2-propandiol)** | -1.57E+08 | 2.18E+00 | -3.49E+08 | 1.72E+00 | -2.01E+08 | 1.08E+00 |
| **Hydroxyacetone (acetol)** | **Formic acid** | -1.16E+08 | 3.47E+00 | -2.05E+08 | 2.15E+00 | -6.86E+07 | 1.08E+00 |
| **Ethylene** | **Ethyl acetate** | -8.34E+07 | 2.10E+00 | -1.33E+08 | 1.48E+00 | -1.02E+08 | 1.07E+00 |
| **Formic acid** | **Acrylic acid** | -1.76E+08 | 2.02E+00 | -3.88E+08 | 1.70E+00 | -1.12E+08 | 1.08E+00 |
| **Methane** | **Acrylic acid** | -1.08E+08 | 1.98E+00 | -2.05E+08 | 1.53E+00 | -1.18E+08 | 1.08E+00 |
| **Ethylene** | **Lactic acid (from 1,2-propandiol)** | -1.91E+08 | 2.05E+00 | -3.94E+08 | 1.59E+00 | -2.94E+08 | 1.09E+00 |
| **Ethylene** | **Acetic acid** | -1.01E+08 | 4.29E+00 | -1.87E+08 | 2.66E+00 | -3.90E+07 | 1.07E+00 |
| **Methanol** | **Lactic acid (from 1,2-propandiol)** | -1.49E+08 | 2.02E+00 | -3.08E+08 | 1.58E+00 | -2.66E+08 | 1.10E+00 |
| **Glycolaldehyde** | **Acrylic acid** | -1.28E+08 | 1.98E+00 | -2.54E+08 | 1.61E+00 | -9.98E+07 | 1.10E+00 |
| **Propionaldehyde** | **Acrylic acid** | -1.30E+08 | 2.04E+00 | -2.64E+08 | 1.61E+00 | -1.52E+08 | 1.10E+00 |
| **Methane** | **Lactic acid (from 1,2-propandiol)** | -1.18E+08 | 2.06E+00 | -2.28E+08 | 1.57E+00 | -1.96E+08 | 1.10E+00 |
| **Hydroxyacetone (acetol)** | **Acrylic acid** | -1.33E+08 | 2.03E+00 | -2.87E+08 | 1.61E+00 | -2.55E+08 | 1.11E+00 |
| **Propionaldehyde** | **Oxalic acid** | -1.17E+08 | 2.90E+00 | -2.18E+08 | 1.96E+00 | -1.20E+08 | 1.11E+00 |
| **Acetone** | **Acrylic acid** | -1.20E+08 | 1.95E+00 | -2.10E+08 | 1.55E+00 | -8.57E+07 | 1.11E+00 |
| **Propionaldehyde** | **Ethyl acetate** | -7.70E+07 | 2.23E+00 | -1.24E+08 | 1.54E+00 | -1.30E+08 | 1.11E+00 |
| **Methanol** | **Acrylic acid** | -1.33E+08 | 1.95E+00 | -2.50E+08 | 1.56E+00 | -1.37E+08 | 1.12E+00 |
| **Propionaldehyde** | **Lactic acid (from 1,2-propandiol)** | -1.45E+08 | 2.13E+00 | -3.03E+08 | 1.65E+00 | -2.95E+08 | 1.13E+00 |
| **Ethylene** | **Oxalic acid** | -1.50E+08 | 2.67E+00 | -2.85E+08 | 1.87E+00 | -1.86E+08 | 1.12E+00 |
| **Acetic acid** | **Oxalic acid** | -1.30E+08 | 2.86E+00 | -2.58E+08 | 2.01E+00 | -1.69E+08 | 1.13E+00 |
| **Formic acid** | **Acetaldehyde** | -1.31E+08 | 2.63E+00 | -2.72E+08 | 1.98E+00 | -1.14E+08 | 1.13E+00 |
| **Ethylene** | **Acrylic acid** | -1.60E+08 | 1.97E+00 | -3.07E+08 | 1.58E+00 | -1.96E+08 | 1.14E+00 |
| **Propionaldehyde** | **Formic acid** | -1.12E+08 | 3.80E+00 | -1.94E+08 | 2.25E+00 | -1.10E+08 | 1.14E+00 |
| **Ethylene glycol** | **Acetaldehyde** | -1.06E+08 | 2.72E+00 | -1.91E+08 | 1.84E+00 | -1.68E+08 | 1.15E+00 |
| **Allyl alcohol** | **Hydrogen Peroxide** | -5.25E+07 | 3.18E+00 | -5.94E+07 | 1.99E+00 | -5.37E+06 | 1.06E+00 |
| **Formic acid** | **Acetic acid** | -1.27E+08 | 3.02E+00 | -2.88E+08 | 2.22E+00 | -1.77E+08 | 1.15E+00 |
| **Ethylene** | **Formic acid** | -1.41E+08 | 3.63E+00 | -2.41E+08 | 2.16E+00 | -1.41E+08 | 1.14E+00 |
| **Allyl alcohol** | **Benzoic acid** | -1.88E+08 | 2.47E+00 | -3.87E+08 | 1.83E+00 | -3.91E+08 | 1.17E+00 |
| **Formic acid** | **Benzoic acid** | -2.23E+08 | 2.46E+00 | -5.42E+08 | 1.96E+00 | -5.18E+08 | 1.18E+00 |
| **Acetic acid** | **Acrylic acid** | -1.39E+08 | 2.08E+00 | -2.54E+08 | 1.69E+00 | -1.26E+08 | 1.19E+00 |
| **Ethylene glycol** | **Benzoic acid** | -2.38E+08 | 2.59E+00 | -5.06E+08 | 1.93E+00 | -5.51E+08 | 1.20E+00 |
| **Ethanol** | **Lactic acid (from 1,2-propandiol)** | -1.48E+08 | 2.04E+00 | -2.99E+08 | 1.57E+00 | -5.32E+08 | 1.20E+00 |
| **Ethylene** | **Benzoic acid** | -2.37E+08 | 2.68E+00 | -5.14E+08 | 1.99E+00 | -5.21E+08 | 1.20E+00 |
| **Acetaldehyde** | **Acetaldehyde** | -8.78E+07 | 2.86E+00 | -1.60E+08 | 1.96E+00 | -1.14E+08 | 1.20E+00 |
| **Glycolaldehyde** | **Acetaldehyde** | -9.35E+07 | 2.78E+00 | -1.69E+08 | 1.95E+00 | -1.06E+08 | 1.20E+00 |
| *n-*Propanol | Acetic acid | -8.73E+07 | 3.55E+00 | -1.49E+08 | 2.16E+00 | -1.34E+08 | 1.21E+00 |
| *n-*Propanol | Benzoic acid | -2.13E+08 | 2.58E+00 | -4.46E+08 | 1.90E+00 | -5.70E+08 | 1.23E+00 |
| Hydroxyacetone (acetol) | Acetaldehyde | -9.64E+07 | 3.01E+00 | -1.82E+08 | 2.04E+00 | -2.08E+08 | 1.24E+00 |
| Acetone | Acetaldehyde | -8.46E+07 | 2.82E+00 | -1.42E+08 | 1.93E+00 | -8.82E+07 | 1.22E+00 |
| Acetic acid | Benzoic acid | -2.04E+08 | 2.71E+00 | -4.54E+08 | 2.04E+00 | -5.34E+08 | 1.24E+00 |
| Acetone | Benzoic acid | -1.90E+08 | 2.63E+00 | -4.02E+08 | 1.94E+00 | -5.39E+08 | 1.25E+00 |
| Ethanol | Acetaldehyde | -9.19E+07 | 2.95E+00 | -1.62E+08 | 1.98E+00 | -1.53E+08 | 1.24E+00 |
| Methanol | Acetaldehyde | -8.96E+07 | 2.89E+00 | -1.59E+08 | 1.98E+00 | -1.31E+08 | 1.24E+00 |
| Ethylene glycol | Acetic acid | -1.03E+08 | 3.55E+00 | -1.84E+08 | 2.25E+00 | -1.90E+08 | 1.26E+00 |
| Methane | Acetaldehyde | -8.00E+07 | 3.09E+00 | -1.34E+08 | 1.96E+00 | -1.57E+08 | 1.24E+00 |
| Carbon monoxide | Glycolic acid | -2.06E+08 | 2.37E+00 | -4.90E+08 | 1.90E+00 | -5.54E+08 | 1.23E+00 |
| Formic acid | Hydrogen Peroxide | -8.89E+07 | 3.15E+00 | -1.34E+08 | 2.31E+00 | -3.10E+07 | 1.22E+00 |
| Ethanol (Cascade) | Acetaldehyde (Cascade) | -9.12E+07 | 3.12E+00 | -1.60E+08 | 2.06E+00 | -1.49E+08 | 1.28E+00 |
| Ethanol | Benzoic acid | -2.06E+08 | 2.67E+00 | -4.42E+08 | 1.98E+00 | -6.34E+08 | 1.28E+00 |
| Hydroxyacetone (acetol) | Benzoic acid | -2.01E+08 | 2.69E+00 | -4.39E+08 | 2.01E+00 | -6.13E+08 | 1.28E+00 |
| Methanol | Benzoic acid | -2.09E+08 | 2.67E+00 | -4.47E+08 | 1.97E+00 | -6.47E+08 | 1.28E+00 |
| Methane | Benzoic acid | -1.82E+08 | 2.74E+00 | -3.83E+08 | 2.00E+00 | -5.32E+08 | 1.28E+00 |
| Hydroxyacetone (acetol) | Acetic acid | -9.31E+07 | 4.05E+00 | -1.77E+08 | 2.58E+00 | -1.59E+08 | 1.28E+00 |
| Ethylene glycol | Hydrogen Peroxide | -6.53E+07 | 3.81E+00 | -7.74E+07 | 2.38E+00 | -1.48E+07 | 1.20E+00 |
| Propionaldehyde | Benzoic acid | -2.02E+08 | 2.75E+00 | -4.33E+08 | 2.02E+00 | -6.29E+08 | 1.30E+00 |
| Ethylene | Acetaldehyde | -1.03E+08 | 2.97E+00 | -1.90E+08 | 2.05E+00 | -1.78E+08 | 1.28E+00 |
| Ethanol | Acetic acid | -8.94E+07 | 4.12E+00 | -1.63E+08 | 2.54E+00 | -1.52E+08 | 1.29E+00 |
| Propionaldehyde | Acetaldehyde | -9.53E+07 | 3.16E+00 | -1.79E+08 | 2.12E+00 | -2.06E+08 | 1.30E+00 |
| Acetone | Acetic acid | -8.23E+07 | 3.79E+00 | -1.55E+08 | 2.43E+00 | -1.65E+08 | 1.30E+00 |
| Allyl alcohol | Benzaldehyde | -2.81E+08 | 2.39E+00 | -8.00E+08 | 2.13E+00 | -8.85E+08 | 1.32E+00 |
| Carbon monoxide | Acrylic acid | -2.15E+08 | 2.53E+00 | -4.93E+08 | 2.15E+00 | -2.73E+08 | 1.29E+00 |
| Acetaldehyde | Acetic acid | -8.50E+07 | 3.83E+00 | -1.58E+08 | 2.40E+00 | -1.84E+08 | 1.32E+00 |
| Ethanol (Cascade) | Acetic acid (Cascade) | -8.87E+07 | 4.63E+00 | -1.60E+08 | 2.79E+00 | -1.50E+08 | 1.35E+00 |
| Acetic acid | Acetaldehyde | -1.00E+08 | 3.06E+00 | -1.79E+08 | 2.18E+00 | -1.19E+08 | 1.35E+00 |
| *n-*Propanol | Hydrogen Peroxide | -5.70E+07 | 3.85E+00 | -6.40E+07 | 2.30E+00 | -1.81E+07 | 1.27E+00 |
| Methane | Acetic acid | -7.68E+07 | 4.51E+00 | -1.32E+08 | 2.64E+00 | -1.39E+08 | 1.34E+00 |
| Formic acid | Formaldehyde | -2.06E+08 | 3.76E+00 | -3.73E+08 | 2.54E+00 | -1.63E+08 | 1.39E+00 |
| Methanol | Acetic acid | -8.72E+07 | 4.06E+00 | -1.63E+08 | 2.56E+00 | -2.10E+08 | 1.40E+00 |
| Formic acid | Benzaldehyde | -3.33E+08 | 2.46E+00 | -9.03E+08 | 2.18E+00 | -9.40E+08 | 1.42E+00 |
| Acetic acid | Ethyl acetate | -8.64E+07 | 2.29E+00 | -1.52E+08 | 1.64E+00 | -4.32E+08 | 1.42E+00 |
| Ethylene glycol | Benzaldehyde | -3.57E+08 | 2.47E+00 | -1.06E+09 | 2.20E+00 | -2.10E+09 | 1.47E+00 |
| Glycolaldehyde | Hydrogen Peroxide | -6.30E+07 | 3.78E+00 | -8.06E+07 | 2.46E+00 | -2.82E+07 | 1.37E+00 |
| *n-*Propanol | Benzaldehyde | -3.17E+08 | 2.44E+00 | -9.11E+08 | 2.19E+00 | -1.33E+09 | 1.47E+00 |
| Carbon monoxide | Acetic acid | -1.55E+08 | 5.04E+00 | -3.91E+08 | 3.80E+00 | -2.17E+08 | 1.37E+00 |
| Carbon monoxide | Benzoic acid | -2.51E+08 | 2.96E+00 | -6.55E+08 | 2.40E+00 | -1.00E+09 | 1.45E+00 |
| Glycolaldehyde | Benzaldehyde | -2.84E+08 | 2.49E+00 | -7.74E+08 | 2.22E+00 | -8.97E+08 | 1.48E+00 |
| Acetic acid | Acetic acid | -9.83E+07 | 4.10E+00 | -2.00E+08 | 2.72E+00 | -2.80E+08 | 1.48E+00 |
| Acetaldehyde | Benzaldehyde | -2.84E+08 | 2.50E+00 | -8.02E+08 | 2.23E+00 | -1.09E+09 | 1.49E+00 |
| Acetaldehyde | Hydrogen Peroxide | -5.64E+07 | 4.11E+00 | -7.04E+07 | 2.54E+00 | -2.43E+07 | 1.38E+00 |
| Acetone | Benzaldehyde | -2.80E+08 | 2.49E+00 | -7.38E+08 | 2.23E+00 | -7.44E+08 | 1.50E+00 |
| Propionaldehyde | Benzaldehyde | -2.96E+08 | 2.56E+00 | -8.62E+08 | 2.28E+00 | -1.44E+09 | 1.50E+00 |
| Hydroxyacetone (acetol) | Benzaldehyde | -2.95E+08 | 2.53E+00 | -8.86E+08 | 2.26E+00 | -1.79E+09 | 1.51E+00 |
| Methane | Benzaldehyde | -2.65E+08 | 2.54E+00 | -7.76E+08 | 2.25E+00 | -1.45E+09 | 1.50E+00 |
| Ethanol | Benzaldehyde | -3.05E+08 | 2.51E+00 | -8.78E+08 | 2.25E+00 | -1.31E+09 | 1.51E+00 |
| Methanol | Benzaldehyde | -3.10E+08 | 2.50E+00 | -8.68E+08 | 2.25E+00 | -1.12E+09 | 1.51E+00 |
| Ethylene | Formaldehyde | -2.03E+08 | 1.45E+01 | -3.02E+08 | 4.43E+00 | -6.05E+07 | 1.43E+00 |
| Ethylene glycol | Oxalic acid | -1.55E+08 | 2.43E+00 | -2.68E+08 | 1.68E+00 | -1.03E+09 | 1.52E+00 |
| Propionaldehyde | Acetic acid | -9.30E+07 | 4.50E+00 | -1.75E+08 | 2.79E+00 | -2.52E+08 | 1.51E+00 |
| Ethylene | Benzaldehyde | -3.59E+08 | 2.51E+00 | -1.02E+09 | 2.26E+00 | -1.43E+09 | 1.52E+00 |
| Acetic acid | Benzaldehyde | -2.98E+08 | 2.56E+00 | -7.51E+08 | 2.30E+00 | -6.65E+08 | 1.53E+00 |
| Acetone | Hydrogen Peroxide | -5.53E+07 | 4.11E+00 | -6.61E+07 | 2.56E+00 | -2.28E+07 | 1.41E+00 |
| Formic acid | Oxygen | -1.18E+08 | 4.08E+00 | -1.97E+08 | 3.00E+00 | -7.11E+07 | 1.51E+00 |
| Carbon monoxide | Benzaldehyde | -3.66E+08 | 2.81E+00 | -9.94E+08 | 2.53E+00 | -9.86E+08 | 1.59E+00 |
| Acetic acid | Hydrogen Peroxide | -6.73E+07 | 4.48E+00 | -8.92E+07 | 2.99E+00 | -3.03E+07 | 1.53E+00 |
| Ethanol | Hydrogen Peroxide | -5.80E+07 | 4.48E+00 | -7.16E+07 | 2.77E+00 | -2.91E+07 | 1.53E+00 |
| Ethylene glycol (Cascade) | Oxalic acid (Cascade) | -1.50E+08 | 2.82E+00 | -2.52E+08 | 1.84E+00 | -1.00E+09 | 1.68E+00 |
| Methanol | Hydrogen Peroxide | -5.66E+07 | 4.47E+00 | -7.12E+07 | 2.83E+00 | -2.94E+07 | 1.56E+00 |
| Methane | Hydrogen Peroxide | -5.09E+07 | 4.97E+00 | -6.11E+07 | 2.88E+00 | -2.45E+07 | 1.52E+00 |
| Methane | Formaldehyde | -1.26E+08 | 1.94E+01 | -2.06E+08 | 4.63E+00 | -8.16E+07 | 1.63E+00 |
| Ethylene | Hydrogen Peroxide | -6.17E+07 | 4.69E+00 | -7.69E+07 | 2.97E+00 | -3.05E+07 | 1.60E+00 |
| Carbon monoxide | Acetaldehyde | -1.60E+08 | 3.86E+00 | -3.53E+08 | 2.93E+00 | -3.59E+08 | 1.73E+00 |
| Propionaldehyde | Formaldehyde | -1.57E+08 | 1.29E+01 | -2.70E+08 | 4.49E+00 | -1.22E+08 | 1.76E+00 |
| Ethylene glycol | Formaldehyde | -2.02E+08 | 6.93E+00 | -3.17E+08 | 3.12E+00 | -2.29E+08 | 1.78E+00 |
| Carbon monoxide | Lactic acid (from 1,2-propandiol) | -2.54E+08 | 2.72E+00 | -6.66E+08 | 2.24E+00 | -2.12E+09 | 1.89E+00 |
| Formic acid | Acetone | -2.09E+08 | 3.95E+00 | -5.56E+08 | 3.17E+00 | -9.74E+08 | 1.94E+00 |
| Allyl alcohol | Oxygen | -7.58E+07 | 6.03E+00 | -1.01E+08 | 3.57E+00 | -4.96E+07 | 1.85E+00 |
| Hydroxyacetone (acetol) | Formaldehyde | -1.56E+08 | 8.85E+00 | -3.18E+08 | 3.73E+00 | -5.77E+08 | 2.01E+00 |
| Acetone | Formaldehyde | -1.43E+08 | 8.79E+00 | -2.06E+08 | 3.57E+00 | -1.37E+08 | 2.11E+00 |
| Acetaldehyde | Benzoic acid | -1.92E+08 | 2.63E+00 | -4.10E+08 | 1.95E+00 | -2.56E+09 | 2.18E+00 |
| Carbon monoxide | Oxalic acid | -2.35E+08 | 3.81E+00 | -6.20E+08 | 3.05E+00 | -1.28E+09 | 2.10E+00 |
| *n-*Propanol | Oxygen | -8.47E+07 | 1.11E+01 | -1.17E+08 | 6.21E+00 | -2.99E+07 | 1.96E+00 |
| *n-*Propanol | Formaldehyde | -1.62E+08 | 7.84E+00 | -2.42E+08 | 3.13E+00 | -2.54E+08 | 2.25E+00 |
| Glycolaldehyde | Formaldehyde | -1.52E+08 | 6.15E+00 | -2.45E+08 | 3.16E+00 | -2.52E+08 | 2.27E+00 |
| Glycolaldehyde | Benzoic acid | -1.91E+08 | 2.58E+00 | -4.18E+08 | 1.95E+00 | -2.94E+09 | 2.33E+00 |
| Acetic acid | Formaldehyde | -1.66E+08 | 8.49E+00 | -2.54E+08 | 4.02E+00 | -1.56E+08 | 2.31E+00 |
| Ethanol | Formaldehyde | -1.59E+08 | 1.14E+01 | -2.58E+08 | 4.10E+00 | -2.06E+08 | 2.34E+00 |
| Carbon monoxide | Hydrogen Peroxide | -1.08E+08 | 5.50E+00 | -1.77E+08 | 4.13E+00 | -9.15E+07 | 2.22E+00 |
| Methanol | Ethyl acetate | -7.14E+07 | 2.05E+00 | -1.08E+08 | 1.44E+00 | -1.86E+09 | 2.47E+00 |
| Methanol | Formaldehyde | -1.61E+08 | 1.22E+01 | -2.51E+08 | 4.20E+00 | -1.93E+08 | 2.44E+00 |
| Acetaldehyde | Formaldehyde | -1.45E+08 | 8.57E+00 | -2.31E+08 | 3.48E+00 | -2.60E+08 | 2.50E+00 |
| Carbon monoxide | Lactic acid (from glycerol) | -1.85E+08 | 1.67E+00 | -3.98E+08 | 1.43E+00 | -4.47E+09 | 2.72E+00 |
| Glycolaldehyde | Acetone | -1.68E+08 | 4.98E+00 | -4.38E+08 | 3.94E+00 | -1.05E+09 | 2.77E+00 |
| Ethylene glycol | Acetone | -2.03E+08 | 5.17E+00 | -5.46E+08 | 4.07E+00 | -1.62E+09 | 2.81E+00 |
| Ethylene glycol | Oxygen | -9.88E+07 | 9.10E+00 | -1.36E+08 | 5.49E+00 | -7.77E+07 | 2.83E+00 |
| Carbon monoxide | Formaldehyde | -2.59E+08 | 8.43E+00 | -5.08E+08 | 5.24E+00 | -3.45E+08 | 2.86E+00 |
| Acetaldehyde | Acetone | -1.67E+08 | 5.60E+00 | -4.46E+08 | 4.29E+00 | -1.39E+09 | 3.07E+00 |
| Acetic acid | Acetone | -1.79E+08 | 5.71E+00 | -4.35E+08 | 4.54E+00 | -6.74E+08 | 3.10E+00 |
| Acetone | Acetone | -1.63E+08 | 5.56E+00 | -4.14E+08 | 4.41E+00 | -8.19E+08 | 3.15E+00 |
| Hydroxyacetone (acetol) | Acetone | -1.76E+08 | 5.76E+00 | -4.78E+08 | 4.54E+00 | -1.47E+09 | 3.17E+00 |
| *n-*Propanol | Acetone | -1.80E+08 | 5.31E+00 | -4.79E+08 | 4.09E+00 | -1.72E+09 | 3.20E+00 |
| Acetaldehyde | Oxygen | -8.11E+07 | 1.22E+01 | -1.19E+08 | 6.86E+00 | -6.01E+07 | 3.05E+00 |
| Ethanol | Acetone | -1.76E+08 | 5.95E+00 | -4.74E+08 | 4.66E+00 | -1.55E+09 | 3.37E+00 |
| Methanol | Acetone | -1.79E+08 | 6.05E+00 | -4.77E+08 | 4.74E+00 | -1.44E+09 | 3.41E+00 |
| Hydroxyacetone (acetol) | Oxygen | -8.99E+07 | 1.29E+01 | -1.47E+08 | 7.93E+00 | -8.91E+07 | 3.36E+00 |
| Methanol (Cascade) | Formaldehyde (Cascade) | -1.60E+08 | 2.06E+01 | -2.57E+08 | 5.98E+00 | -2.10E+08 | 3.59E+00 |
| Carbon monoxide | Acetone | -2.40E+08 | 6.32E+00 | -6.52E+08 | 5.22E+00 | -1.27E+09 | 3.57E+00 |
| Methane | Acetone | -1.54E+08 | 6.58E+00 | -4.08E+08 | 5.06E+00 | -1.35E+09 | 3.67E+00 |
| Propionaldehyde | Acetone | -1.73E+08 | 6.27E+00 | -4.68E+08 | 4.88E+00 | -1.69E+09 | 3.78E+00 |
| Acetic acid | Oxygen | -9.50E+07 | 1.17E+01 | -1.38E+08 | 7.62E+00 | -7.02E+07 | 3.59E+00 |
| Ethylene | Acetone | -2.03E+08 | 6.23E+00 | -5.53E+08 | 4.91E+00 | -2.04E+09 | 3.87E+00 |
| Acetone | Oxygen | -7.97E+07 | 1.26E+01 | -1.13E+08 | 7.74E+00 | -6.52E+07 | 3.88E+00 |
| Carbon monoxide | Formic acid | -2.17E+08 | 4.55E+00 | -5.51E+08 | 3.45E+00 | -2.83E+09 | 4.43E+00 |
| Carbon monoxide | Oxygen | -1.44E+08 | 1.07E+01 | -2.55E+08 | 8.14E+00 | -1.57E+08 | 4.24E+00 |
| Allyl alcohol | Acetone | -1.61E+08 | 4.56E+00 | -4.24E+08 | 3.55E+00 | -3.14E+09 | 4.76E+00 |
| Ethanol | Oxygen | -8.59E+07 | 2.05E+01 | -1.25E+08 | 1.22E+01 | -8.48E+07 | 6.36E+00 |
| Methanol | Oxygen | -8.42E+07 | 2.39E+01 | -1.20E+08 | 1.44E+01 | -7.87E+07 | 6.90E+00 |
| Propionaldehyde | Oxygen | -8.78E+07 | 2.47E+01 | -1.35E+08 | 1.46E+01 | -9.28E+07 | 7.23E+00 |
| Ethylene | Oxygen | -9.61E+07 | 3.77E+01 | -1.33E+08 | 2.21E+01 | -8.78E+07 | 1.15E+01 |
| Methane | Oxygen | -7.42E+07 | 1.04E+02 | -1.06E+08 | 5.72E+01 | -5.11E+07 | 1.85E+01 |

**Supplementary Table 10** Cash flow for CO/2-furoic acid coproduction process

| **Year** | **C_TDC_** | **C_WC_** | **D** | **C_Excl. Dep_** | **S** | **Net Earnings** | **DCF** | **PV** | **Cum. PV** |
| --- | --- | --- | --- | --- | --- | --- | --- | --- | --- |
| **1** | ($12,345,509) | $0 | $0 | $0 | $311,720,709 | $0 | ($12,345,509) | ($10,735,226) | ($10,735,226) |
| **2** | ($12,345,509) | ($33,446,957) | $0 | $0 | $311,720,709 | $0 | ($45,792,467) | ($34,625,684) | ($45,360,909) |
| **3** | $0 | $0 | $282,268 | $165,997,750 | $311,720,709 | $88,864,262 | $89,146,530 | $58,615,290 | $13,254,381 |
| **4** | $0 | $0 | $483,746 | $164,116,274 | $311,720,709 | $89,890,741 | $90,374,487 | $51,671,906 | $64,926,288 |
| **5** | $0 | $0 | $345,477 | $164,116,274 | $311,720,709 | $89,975,223 | $90,320,700 | $44,905,351 | $109,831,638 |
| **6** | $0 | $0 | $246,713 | $164,116,274 | $311,720,709 | $90,035,568 | $90,282,281 | $39,031,521 | $148,863,160 |
| **7** | $0 | $0 | $176,393 | $164,116,274 | $311,720,709 | $90,078,534 | $90,254,927 | $33,930,170 | $182,793,330 |
| **8** | $0 | $0 | $176,195 | $164,116,274 | $311,720,709 | $90,078,655 | $90,254,850 | $29,504,470 | $212,297,800 |
| **9** | $0 | $0 | $176,393 | $164,116,274 | $311,720,709 | $90,078,534 | $90,254,927 | $25,656,083 | $237,953,883 |
| **10** | $0 | $0 | $88,098 | $165,997,750 | $311,720,709 | $88,982,900 | $89,070,998 | $22,016,988 | $259,970,872 |
| **11** | $0 | $0 | $0 | $164,116,274 | $311,720,709 | $90,186,310 | $90,186,310 | $19,384,936 | $279,355,808 |
| **12** | $0 | $0 | $0 | $164,116,274 | $311,720,709 | $90,186,310 | $90,186,310 | $16,856,466 | $296,212,274 |
| **13** | $0 | $0 | $0 | $164,116,274 | $311,720,709 | $90,186,310 | $90,186,310 | $14,657,797 | $310,870,071 |
| **14** | $0 | $0 | $0 | $164,116,274 | $311,720,709 | $90,186,310 | $90,186,310 | $12,745,910 | $323,615,981 |
| **15** | $0 | $33,446,957 | $0 | $164,116,274 | $311,720,709 | $90,186,310 | $123,633,267 | $15,193,847 | $338,809,827 |

**Supplementary Figures**

**
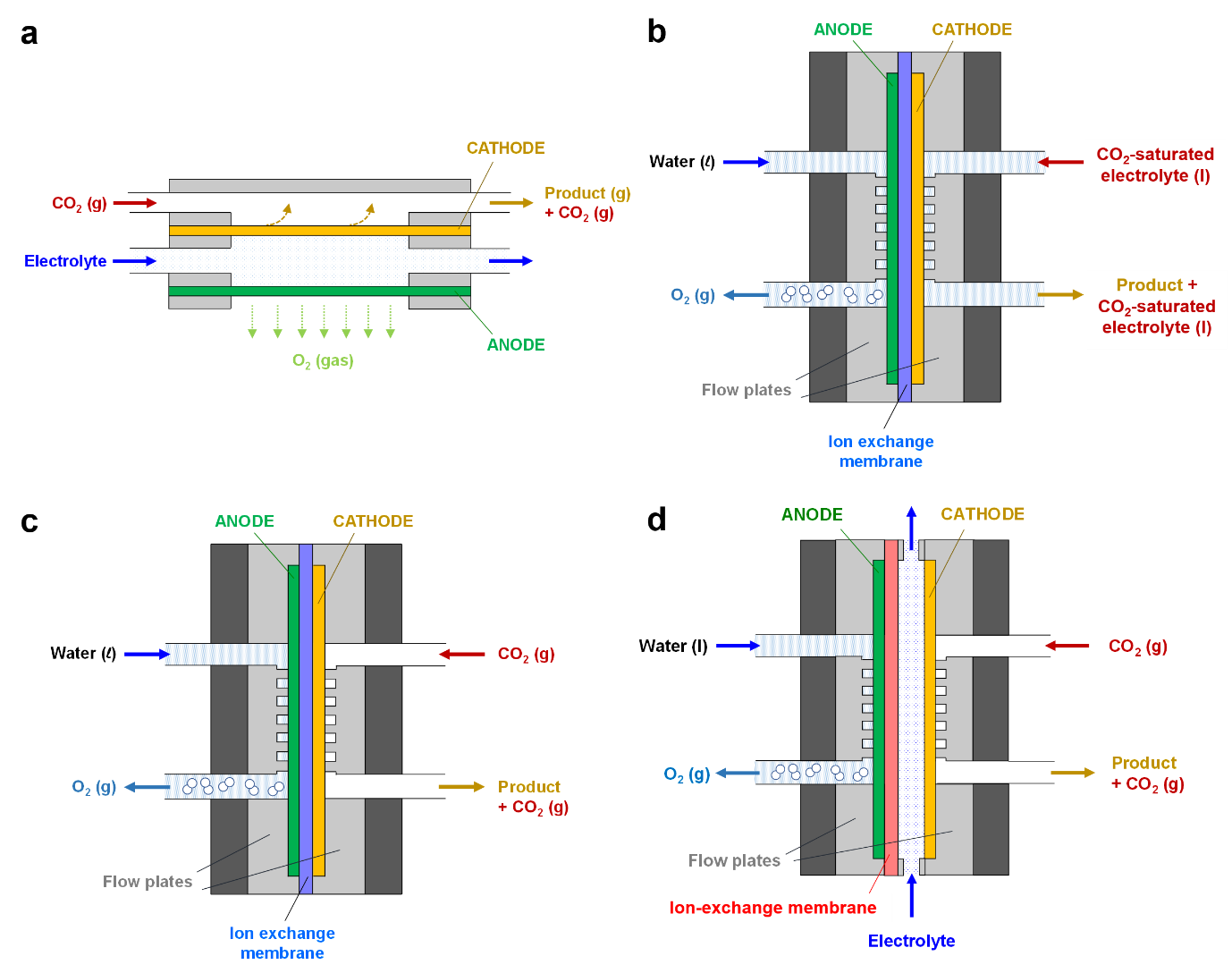
**

Supplementary Figure 1 Schematic representation of different electrochemical reactors for CO_2_RR or water splitting. a, Classical microfluidic flow reactor. b, General design of an electrolyzer with an ion-exchange membrane. Water- or CO_2_-saturated electrolytes are directly supplied to the cathode. c, Humidified CO_2_ supplied to the cathode. d, Representative design of a water electrolyzer with an electrolyte layer between the cathode catalyst layer and an ion-exchange membrane.


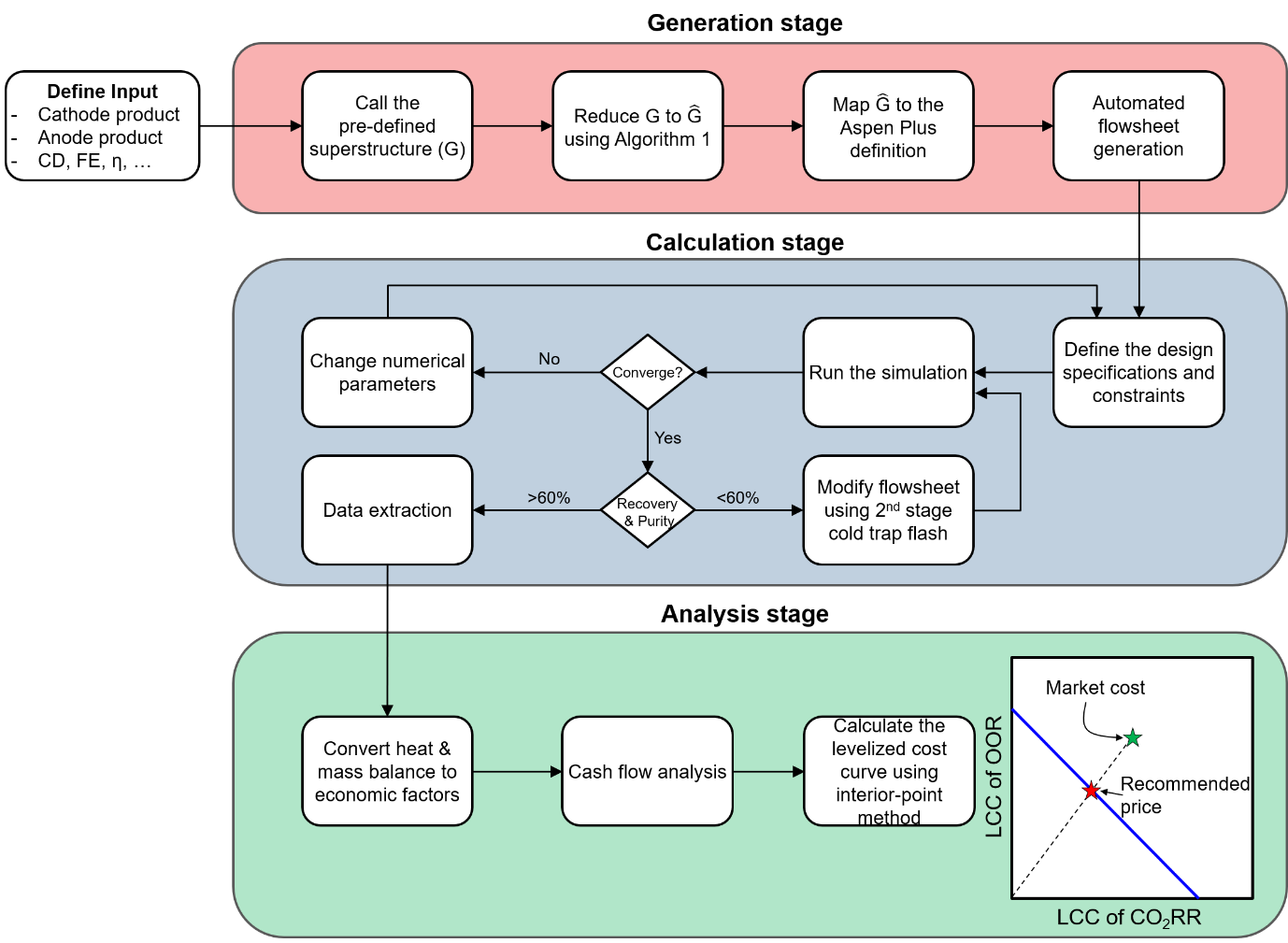


**Supplementary Figure 2 A brief flowchart representing the automatic technoeconomic analysis platform.** Dealing with a flowsheet generation, a flowsheet calculation, and a technoeconomic analysis regarding the levelized cost curve.


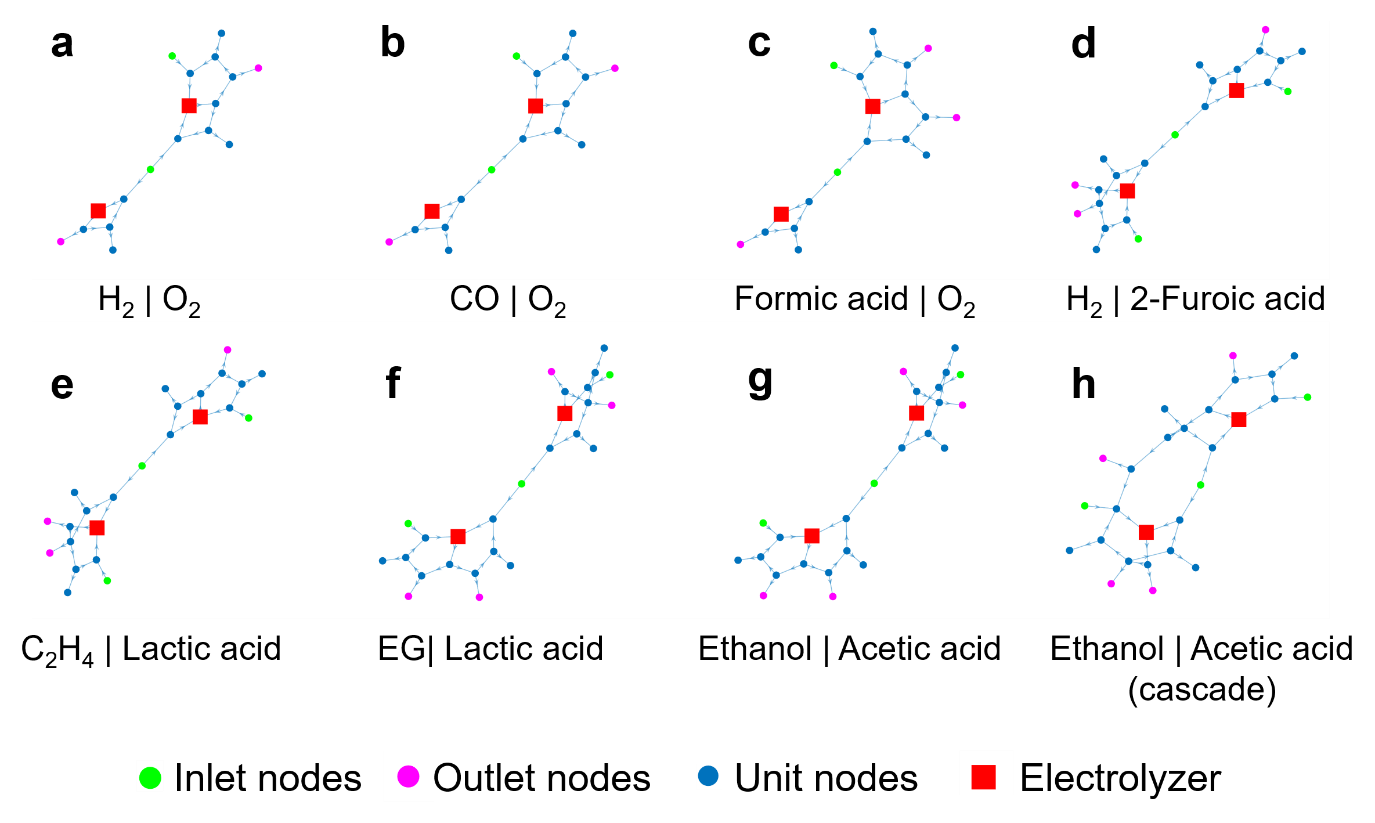


**Supplementary Figure 3 The representative reduced structure for the e-chemical of several combinations.** **a**, HER/OER. **b**, Gas/OER. **c**, Liquid/OER. **d**, HER/liquid. **e**, Gas/liquid. **f**, Liquid/liquid. **g**, Liquid/liquid (non*-*cascade). **h**, Liquid/liquid (cascade).


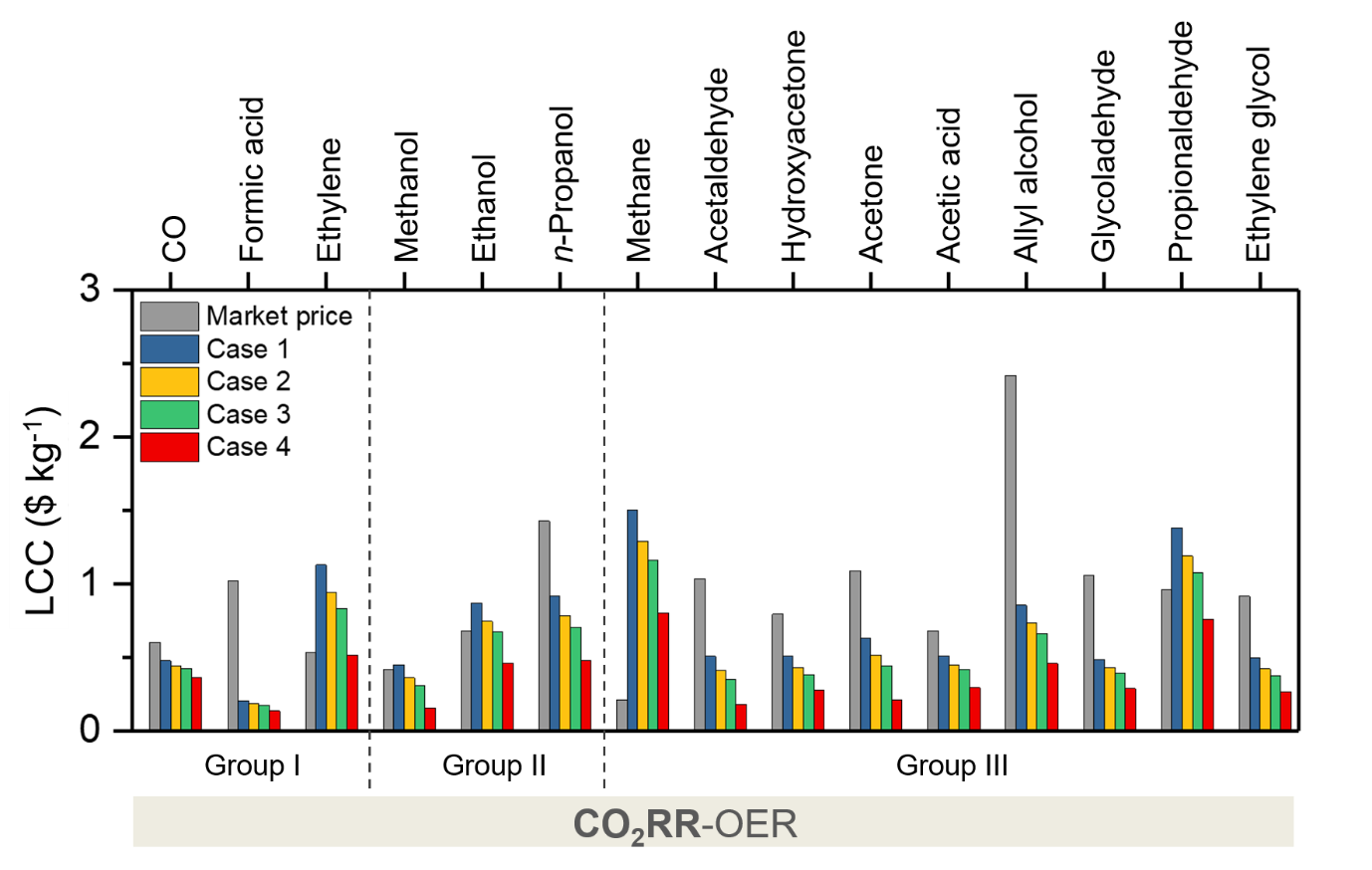


**Supplementary Figure 4 Case study of the electrochemical CO_2_RR–OER coproduction process.** **Case 1** is based on optimal case II in Supplementary Table 9 + 100% recycle of electrolyte, no labor-related cost, and electricity cost (Levelized cost of energy (LCOE) of subsidized solar PV in 2018^238^) as 0.032 $ kWh^-1^. **Case 2** is based on Case 1 + 50% of electrolyzer cost. **Case 3** is based on Case 2 + 20% of electrolyzer cost. **Case 4** is based on Case 3 + electricity cost (Levelized cost of energy (LCOE) of subsidized onshore wind in 2018^238^) as 0.014 $ kWh^-1^.

**
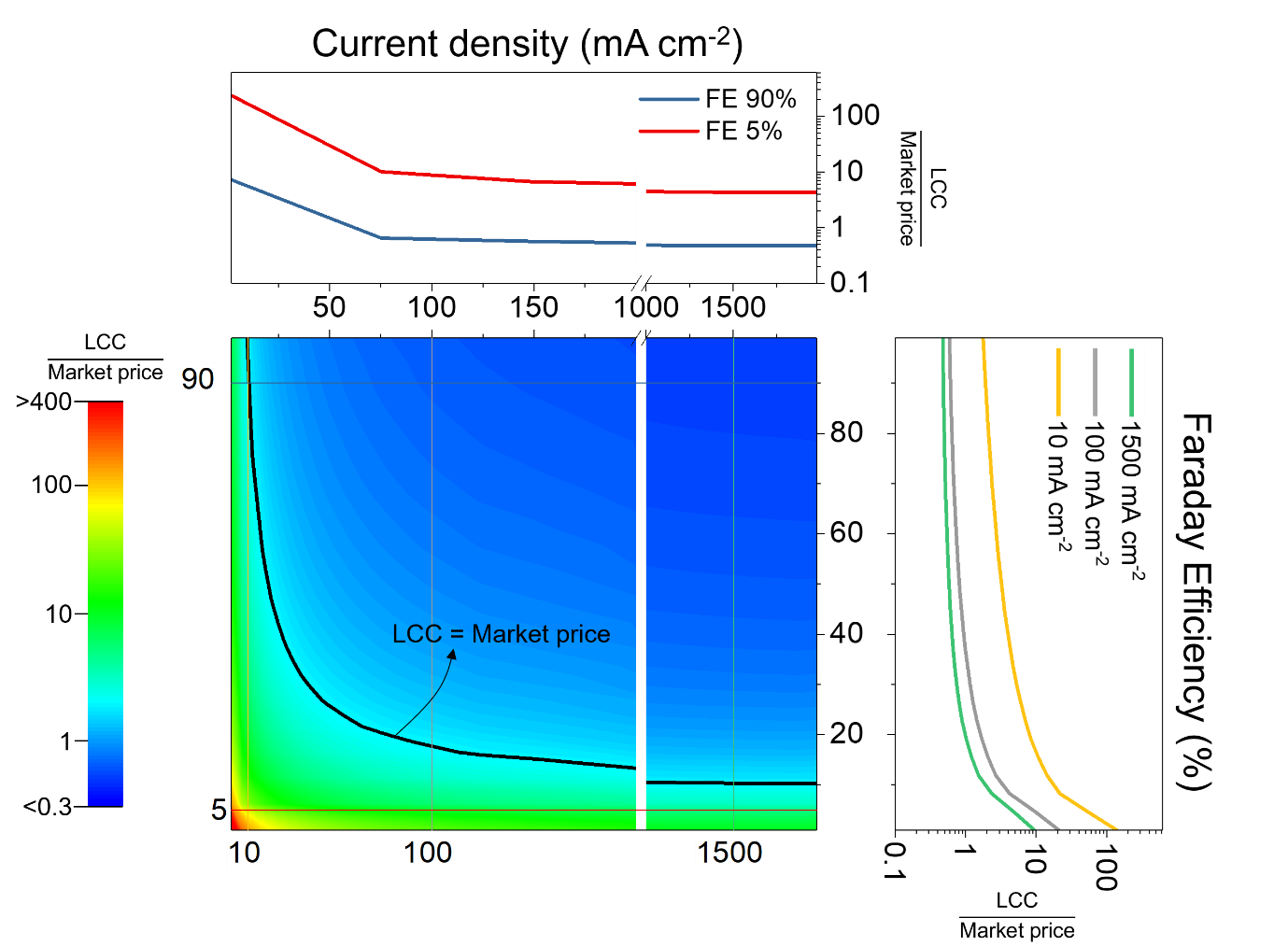
**

**Supplementary Figure 5 A contour plot of the levelized cost over market price ratio for the CO/2-furoic acid electrochemical process as a function of the current density and Faraday efficiency.** Ratio exponentially increases is below Current density 75 mA cm^-2^ and when Faraday efficiency is below 10%. Upper right side of black line is economically feasible that CO/2-furoic acid process should be targeted. Since this black line can be located in different position for different combination of CO2RR–OOR, target current density and Faraday efficiency can be different for different combination.


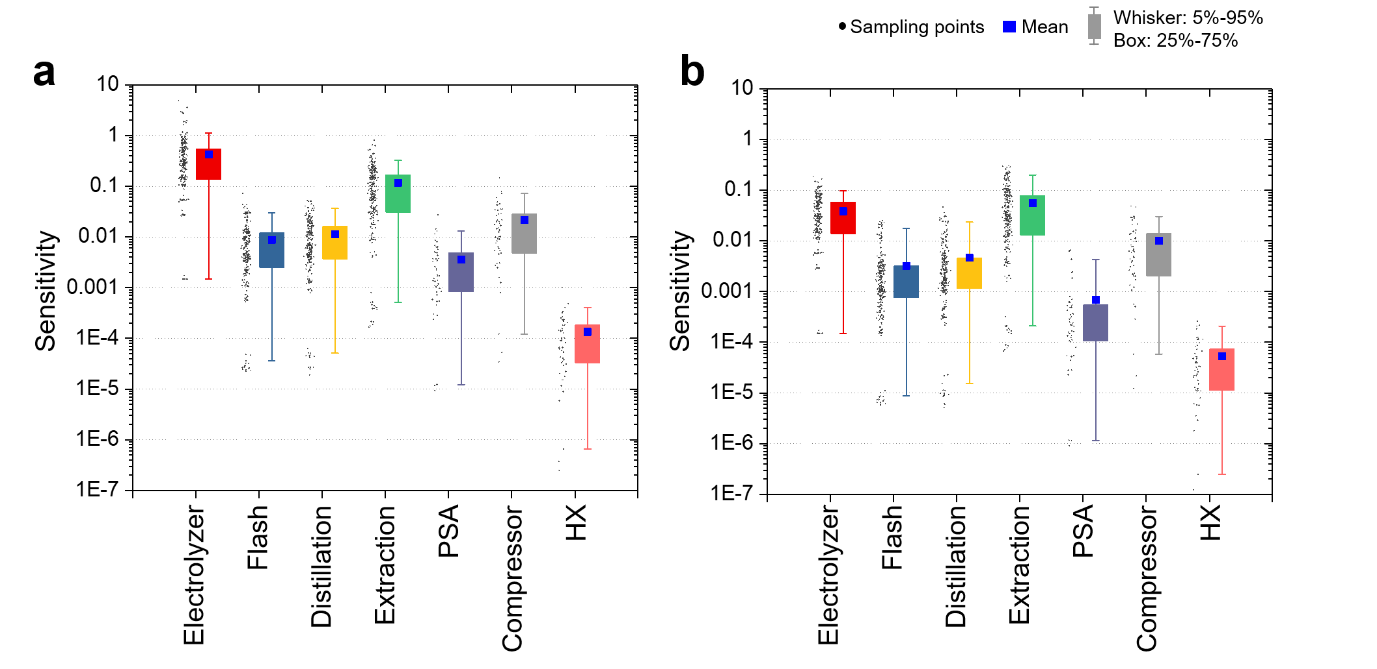


**Supplementary Figure 6 Sensitivity analysis of capital cost variations for every CO_2_RR–OOR processes.** **a**, Based on base case parameters. **b**, Based on optimal case II parameters (Supplementary Table 9). The definition of sensitivity for a unit i is given by,

$$\mathbf{sensitivity}_{\mathbf{i}}\mathbf{=}\frac{\boldsymbol{\partial(}\frac{\mathbf{LCC}}{\mathbf{Market prices}}\mathbf{)}}{\boldsymbol{\partial}\mathbf{(}\mathbf{ratio}_{\mathbf{equipment cost}}\mathbf{)}}$$

where ratio_equipment_ represents equipment cost changed ratio compared to the equipment cost evaluated by the proposed shortcut electrolyzer and separation models. For example, the meaning of sensitivity_PSA_ equals to 0.5 is that if equipment cost of PSA increases by 100%, then LCC increases by 50%.


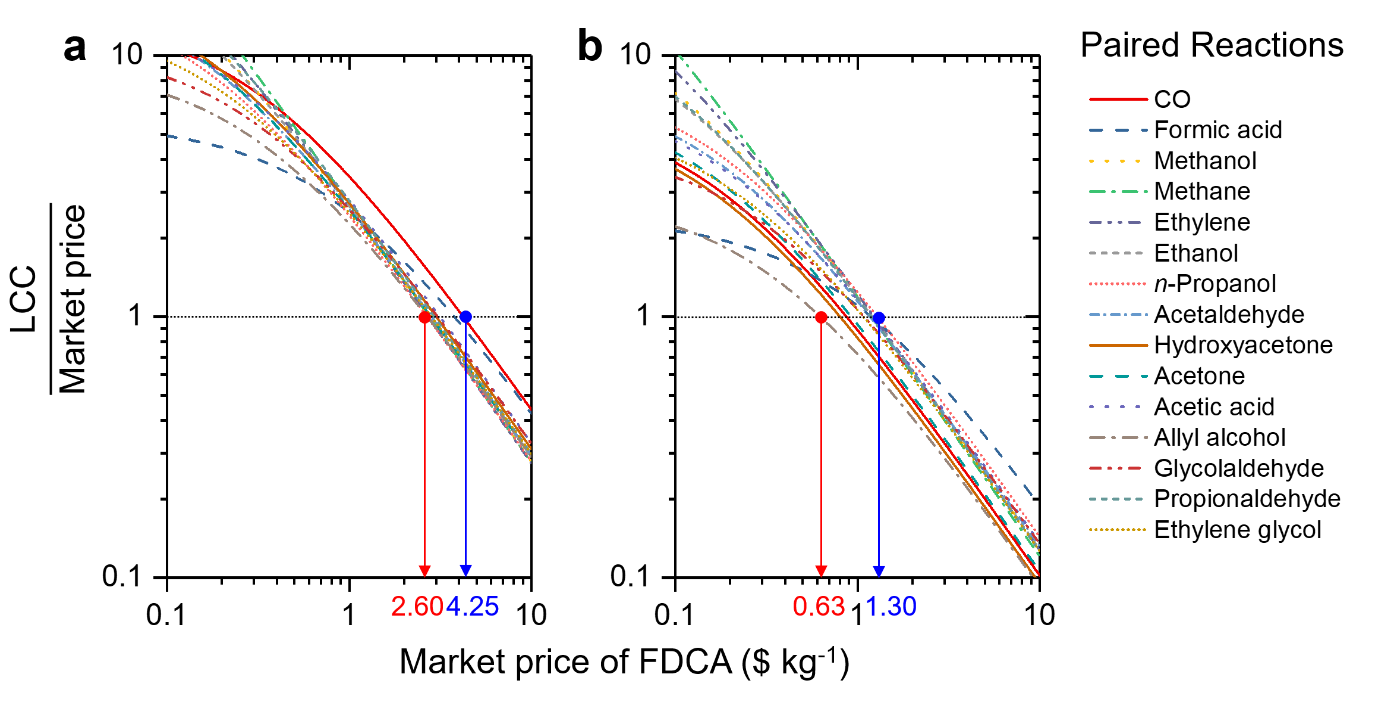


Supplementary Figure 7 Sensitivity of LCC over market price through variation of FDCA market price. a, Based on base case parameters. b, Based on optimal case II parameters (Supplementary Table 9). When the market price of FDCA is over $4.25 kg^-1^ at base case and $1.30 kg^-1^ at optimal case, CO_2_RR–OOR process can secure the economic feasibility, regardless of the CO_2_RR products.


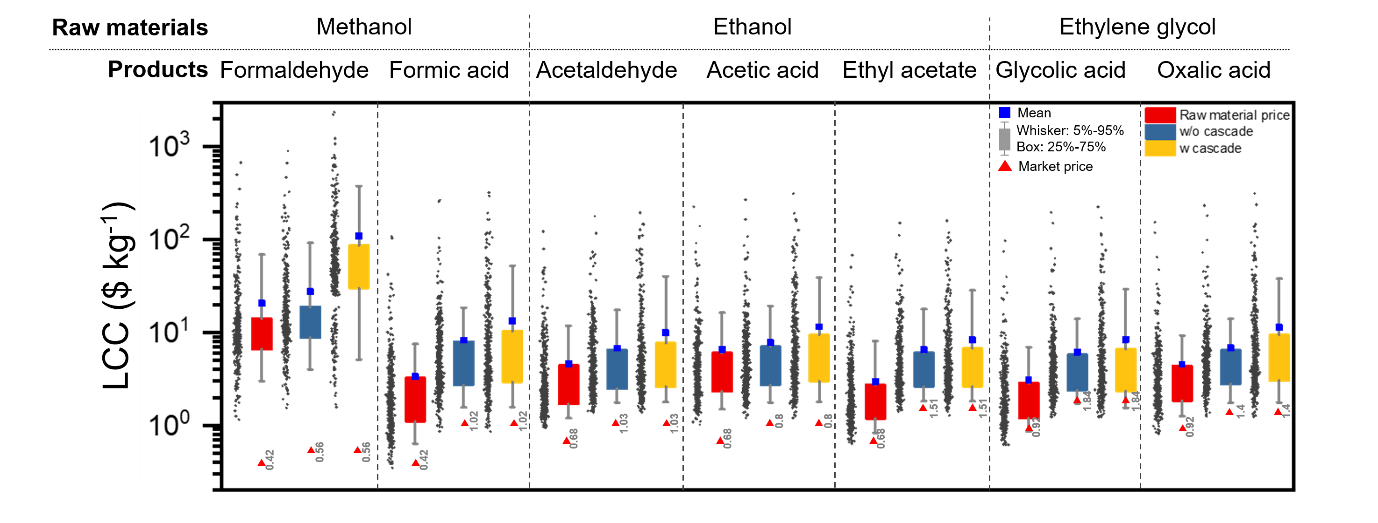


Supplementary Figure 8 LCC distribution for all possible combinations of cascade coproduction processes. The raw material price means the LCC of cathode products (methanol, ethanol and ethylene glycol) without cascade.


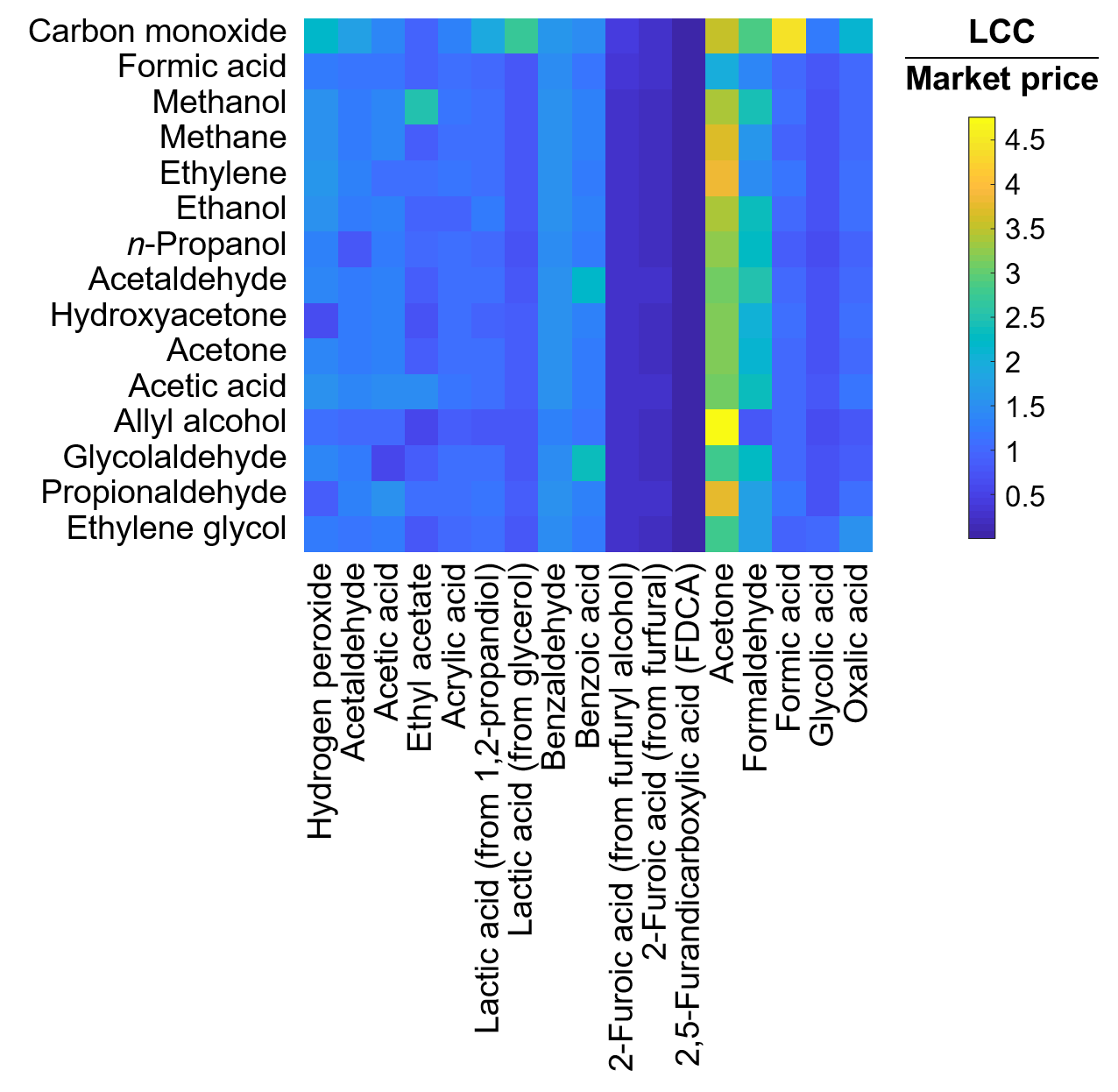


Supplementary Figure 9 Heat map of the LCC-to-market price ratio to visualize the economic feasibility of the optimal case in Supplementary Table 9.

**
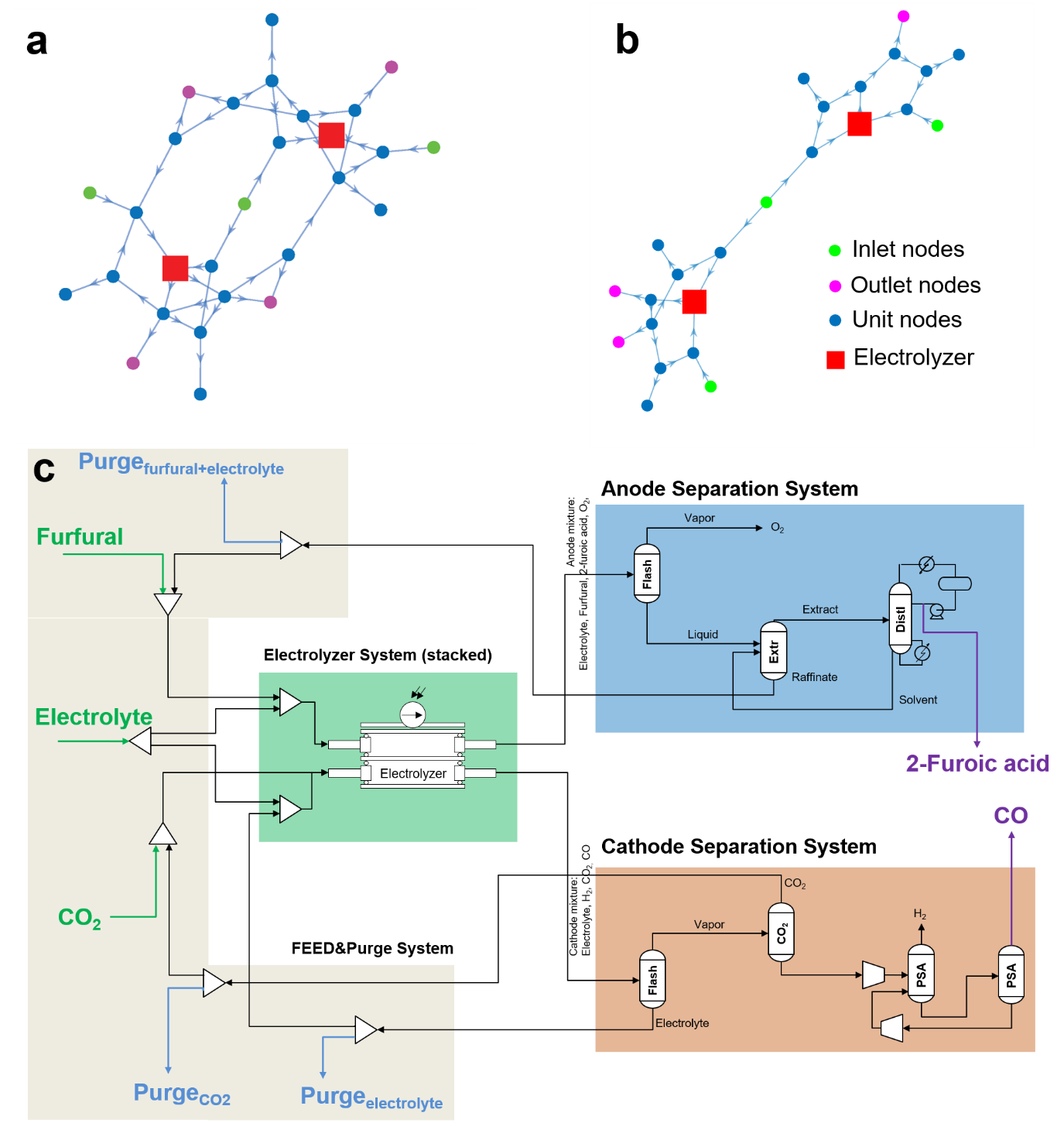
Supplementary Figure 10 Schematic network of the e-chemical process.** **a**, Superstructure. **b**, Reduced structure for CO/2-furoic acid process. **c**, Process flow diagram of CO/2-furoic acid process, which represented the reduced structure (**b**).

**
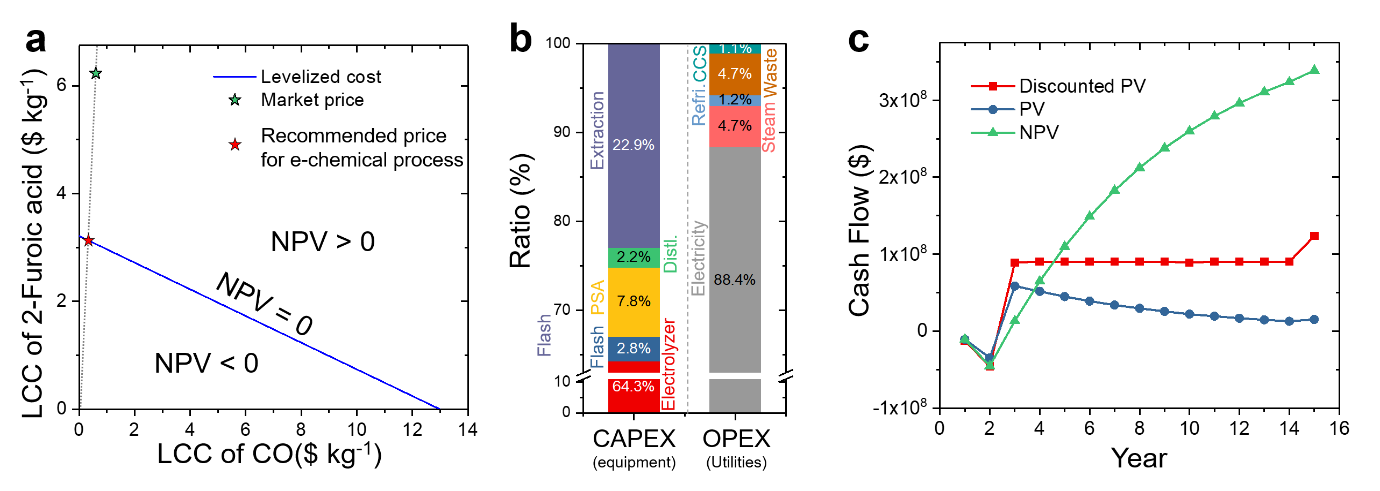
**

**Supplementary Figure 11 The results of technoeconomic analysis for the CO/2-furoic acid electrochemical process.** **a**, The levelized cost line where cumulative NPV equals zero at anywhere on the dotted line. **b,** Contribution of each unit to CAPEX and utility and unit process to OPEX. **c,** NPV cash flow analysis using current market prices of CO and 2-furoic acid.

**Supplementary References**

1 Wang, M., Wang, Z., Gong, X. & Guo, Z. The intensification technologies to water electrolysis for hydrogen production – a review. *Renew. Sustain. Energy Rev.* **29**, 573–588 (2014).

2 Holladay, J. D., Hu, J., King, D. L. & Wang, Y. An overview of hydrogen production technologies. *Catal. Today* **139**, 244–260 (2009).

3 Zhang, L., Zhao, Z.-J. & Gong, J. Nanostructured materials for heterogeneous electrocatalytic CO_2_ reduction and their related reaction mechanisms. *Angew. Chem. Int. Ed.* **56**, 11326–11353 (2017).

4 Lu, Q. & Jiao, F. Electrochemical CO_2_ reduction: electrocatalyst, reaction mechanism, and process engineering. *Nano Energy* **29**, 439–456 (2016).

5 Mota, F. M. & Kim, D. H. From CO_2_ methanation to ambitious long-chain hydrocarbons: alternative fuels paving the path to sustainability. *Chem. Soc. Rev.* **48**, 205–259 (2019).

6 Martín, A. J., Larrazábal, G. O. & Pérez-Ramírez, J. Towards sustainable fuels and chemicals through the electrochemical reduction of CO_2_: lessons from water electrolysis. *Green Chem.* **17**, 5114–5130 (2015).

7 Kortlever, R., Shen, J., Schouten, K. J. P., Calle-Vallejo, F. & Koper, M. T. M. Catalysts and reaction pathways for the electrochemical reduction of carbon dioxide. *J. Phys. Chem. Lett.* **6**, 4073–4082 (2015).

8 Schouten, K. J. P., Kwon, Y., van der Ham, C. J. M., Qin, Z. & Koper, M. T. M. A new mechanism for the selectivity to C_1_ and C_2_ species in the electrochemical reduction of carbon dioxide on copper electrodes. *Chem. Sci.* **2**, 1902–1909 (2011).

9 Khezri, B., Fisher, A. C. & Pumera, M. CO_2_ reduction: the quest for electrocatalytic materials. *J. Mater. Chem. A* **5**, 8230–8246 (2017).

10 Vesborg, P. C. K., Seger, B. & Chorkendorff, I. Recent development in hydrogen evolution reaction catalysts and their practical implementation. *J. Phys. Chem. Lett.* **6**, 951–957 (2015).

11 Mistry, H. *et al.* Highly selective plasma-activated copper catalysts for carbon dioxide reduction to ethylene. *Nat. Commun.* **7**, 12123 (2016).

12 Dinh, C.-T. *et al.* CO_2_ electroreduction to ethylene via hydroxide-mediated copper catalysis at an abrupt interface. *Science* **360**, 783–787 (2018).

13 Liu, Z., Yang, H., Kutz, R. & Masel, R. I. CO_2_ electrolysis to CO and O_2_ at high selectivity, stability and efficiency using sustainion membranes. *J. Electrochem. Soc.* **165**, J3371–J3377 (2018).

14 Neyerlin, K. C., Gu, W., Jorne, J. & Gasteiger, H. A. Study of the exchange current density for the hydrogen oxidation and evolution reactions. *J. Electrochem. Soc.* **154**, B631–B635 (2007).

15 Popczun, E. J., Read, C. G., Roske, C. W., Lewis, N. S. & Schaak, R. E. Highly active electrocatalysis of the hydrogen evolution reaction by cobalt phosphide nanoparticles. *Angew. Chem. Int. Ed.* **53**, 5427–5430 (2014).

16 Wang, H. *et al.* Electrochemical Tuning of MoS_2_ Nanoparticles on Three-Dimensional Substrate for Efficient Hydrogen Evolution. *ACS Nano* **8**, 4940-4947 (2014).

17 Kim, C. *et al.* Insight into electrochemical CO_2_ reduction on surface-molecule-mediated ag nanoparticles. *ACS Catal.* **7**, 779–785 (2017).

18 Zhang, S., Kang, P. & Meyer, T. J. Nanostructured tin catalysts for selective electrochemical reduction of carbon dioxide to formate. *J. Am. Chem. Soc.* **136**, 1734–1737 (2014).

19 Lee, S. Y. *et al.* Mixed copper states in anodized cu electrocatalyst for stable and selective ethylene production from CO_2_ reduction. *J. Am. Chem. Soc.* **140**, 8681–8689 (2018).

20 Kaczur, J. J., Yang, H., Liu, Z., Sajjad, S. D. & Masel, R. I. Carbon dioxide and water electrolysis using new alkaline stable anion membranes. *Front. Chem.* **6**, 263 (2018).

21 Kutz, R. B. *et al.* Sustainion imidazolium-functionalized polymers for carbon dioxide electrolysis. *Energy Technol.* **5**, 929–936 (2017).

22 Kirubakaran, A., Jain, S. & Nema, R. K. A review on fuel cell technologies and power electronic interface. *Renew. Sustain. Energy Rev.* **13**, 2430–2440 (2009).

23 Zhu, Y., Ha, S. Y. & Masel, R. I. High power density direct formic acid fuel cells. *J. Power Sources* **130**, 8–14 (2004).

24 Yu, X. & Pickup, P. G. Recent advances in direct formic acid fuel cells (DFAFC). *J. Power Sources* **182**, 124–132 (2008).

25 Munir, S., Varzeghani, A. R. & Kaya, S. Electrocatalytic reduction of CO_2_ to produce higher alcohols. *Sustain. Energy Fuels* **2**, 2532–2541 (2018).

26 Kuhl, K. P., Cave, E. R., Abram, D. N. & Jaramillo, T. F. New insights into the electrochemical reduction of carbon dioxide on metallic copper surfaces. *Energy Environ. Sci.* **5**, 7050–7059 (2012).

27 Chen, Y. X. *et al.* Nanotechnology makes biomass electrolysis more energy efficient than water electrolysis. *Nat. Commun.* **5**, 4036 (2014).

28 Mahmoudian, J. *et al.* Electrochemical coproduction of acrylate and hydrogen from 1,3-propandiol. *ACS Sustain. Chem. Eng.* **5**, 6090–6098 (2017).

29 Sumit, V., Byoungsu, K., “Molly”, J. H. R., Sichao, M. & A., K. P. J. A Gross‐Margin Model for Defining Technoeconomic Benchmarks in the Electroreduction of CO2. *ChemSusChem* **9**, 1972-1979 (2016).

30 Cha, H. G. & Choi, K.-S. Combined biomass valorization and hydrogen production in a photoelectrochemical cell. *Nat. Chem.* **7**, 328–333 (2015).

31 Rosatella, A. A., Simeonov, S. P., Frade, R. F. M. & Afonso, C. A. M. 5-hydroxymethylfurfural (HMF) as a building block platform: biological properties, synthesis and synthetic applications. *Green Chem.* **13**, 754–793 (2011).

32 Kwon, Y., Schouten, K. J. P., van der Waal, J. C., de Jong, E. & Koper, M. T. M. Electrocatalytic conversion of furanic compounds. *ACS Catal.* **6**, 6704–6717 (2016).

33 You, B., Liu, X., Liu, X. & Sun, Y. Efficient H2 Evolution Coupled with Oxidative Refining of Alcohols via A Hierarchically Porous Nickel Bifunctional Electrocatalyst. *ACS Catalysis* **7**, 4564-4570 (2017).

34 Ma , S., Lan , Y., Perez, G. M. J., Moniri, S. & Kenis , P. J. A. Silver supported on titania as an active catalyst for electrochemical carbon dioxide reduction. *ChemSusChem* **7**, 866–874 (2014).

35 Whipple, D. T., Finke, E. C. & Kenis, P. J. A. Microfluidic reactor for the electrochemical reduction of carbon dioxide: the effect of pH. *Electrochem. Sol. State Lett.* **13**, B109–B111 (2010).

36 Jhong, H.-R. M., Brushett, F. R. & Kenis, P. J. A. The effects of catalyst layer deposition methodology on electrode performance. *Adv. Energy Mater.* **3**, 589–599 (2013).

37 Kim, B., Ma, S., Jhong, H.-R. M. & Kenis, P. J. A. Influence of dilute feed and pH on electrochemical reduction of CO_2_ to CO on Ag in a continuous flow electrolyzer. *Electrochim. Acta* **166**, 271–276 (2015).

38 Barbir, F. PEM electrolysis for production of hydrogen from renewable energy sources. *Sol. Energy* **78**, 661–669 (2005).

39 Vincent, I. & Bessarabov, D. Low cost hydrogen production by anion exchange membrane electrolysis: a review. *Renew. Sustain. Energy Rev.* **81**, 1690–1704 (2018).

40 Babic, U., Suermann, M., Büchi, F. N., Gubler, L. & Schmidt, T. J. Critical review—identifying critical gaps for polymer electrolyte water electrolysis development. *J. Am. Chem. Soc.* **164**, F387–F399 (2017).

41 Marangio, F., Pagani, M., Santarelli, M. & Calì, M. Concept of a high pressure PEM electrolyser prototype. *Int. J. Hydrog. Energy* **36**, 7807–7815 (2011).

42 Bareiß, K., de la Rua, C., Möckl, M. & Hamacher, T. Life cycle assessment of hydrogen from proton exchange membrane water electrolysis in future energy systems. *Appl. Energy* **237**, 862–872 (2019).

43 Higgins, D., Hahn, C., Xiang, C., Jaramillo, T. F. & Weber, A. Z. Gas-diffusion electrodes for carbon dioxide reduction: a new paradigm. *ACS Energy Lett.* **4**, 317–324 (2019).

44 Dufek, E. J., Lister, T. E. & McIlwain, M. E. Bench-scale electrochemical system for generation of CO and syn-gas. *J. Appl. Electrochem.* **41**, 623–631 (2011).

45 Delacourt, C., Ridgway, P. L. & Newman, J. Mathematical modeling of CO_2_ reduction to CO in aqueous electrolytes: I. Kinetic study on planar silver and gold electrodes. *J. Electrochem. Soc.* **157**, B1902–B1910 (2010).

46 Dinh, C.-T., García de Arquer, F. P., Sinton, D. & Sargent, E. H. High rate, selective, and stable electroreduction of CO_2_ to CO in basic and neutral media. *ACS Energy Lett.* **3**, 2835–2840 (2018).

47 Liu, K., Smith, W. A. & Burdyny, T. Introductory guide to assembling and operating gas diffusion electrodes for electrochemical CO_2_ reduction. *ACS Energy Lett.* **4**, 639–643 (2019).

48 You, B. & Sun, Y. Innovative strategies for electrocatalytic water splitting. *Acc. Chem. Res.* **51**, 1571–1580 (2018).

49 Wang, G. *et al.* Energy-efficient electrolytic hydrogen production assisted by coupling urea oxidation with a pH-gradient concentration cell. *Chemical Communications* **54**, 2603-2606 (2018).

50 Jun‐Ye, Z. *et al.* Anodic hydrazine oxidation assists energy‐efficient hydrogen evolution over a bifunctional cobalt perselenide nanosheet electrode. *Angew. Chem.* **130**, 7775–7779 (2018).

51 You, B., Liu, X., Liu, X. & Sun, Y. Efficient H_2_ evolution coupled with oxidative refining of alcohols via a hierarchically porous nickel bifunctional electrocatalyst. *ACS Catal.* **7**, 4564–4570 (2017).

52 Li, T. *et al.* Photoelectrochemical oxidation of organic substrates in organic media. *Nat. Commun.* **8**, 390 (2017).

53 Han, G. *et al.* Visible-light-driven valorization of biomass intermediates integrated with H_2_ production catalyzed by ultrathin Ni/CdS nanosheets. *J. Am. Chem. Soc.* **139**, 15584–15587 (2017).

54 Jiang, N., You, B., Boonstra, R., Terrero Rodriguez, I. M. & Sun, Y. Integrating Electrocatalytic 5-Hydroxymethylfurfural Oxidation and Hydrogen Production via Co–P-Derived Electrocatalysts. *ACS Energy Letters* **1**, 386-390 (2016).

55 Horn, E. J. *et al.* Scalable and sustainable electrochemical allylic C–H oxidation. *Nature* **533**, 77 (2016).

56 Badalyan, A. & Stahl, S. S. Cooperative electrocatalytic alcohol oxidation with electron-proton-transfer mediators. *Nature* **535**, 406–410 (2016).

57 Chen, Y. X., Miki, A., Ye, S., Sakai, H. & Osawa, M. Formate, an active intermediate for direct oxidation of methanol on Pt electrode. *J. Am. Chem. Soc.* **125**, 3680–3681 (2003).

58 Hickey, D. P. *et al.* Predicting electrocatalytic properties: modeling structure–activity relationships of nitroxyl radicals. *J. Am. Chem. Soc.* **137**, 16179–16186 (2015).

59 Motagamwala, A. H. *et al.* Toward biomass-derived renewable plastics: production of 2,5-furandicarboxylic acid from fructose. *Sci. Adv.* **4**, eaap9722 (2018).

60 Sherbo, R. S., Delima, R. S., Chiykowski, V. A., MacLeod, B. P. & Berlinguette, C. P. Complete electron economy by pairing electrolysis with hydrogenation. *Nat. Catal.* **1**, 501–507 (2018).

61 Zhang, Z. & Huber, G. W. Catalytic oxidation of carbohydrates into organic acids and furan chemicals. *Chem. Soc. Rev.* **47**, 1351–1390 (2018).

62 Herron, J. A., Kim, J., Upadhye, A. A., Huber, G. W. & Maravelias, C. T. A general framework for the assessment of solar fuel technologies. *Energy & Environmental Science* **8**, 126-157 (2015).

63 Sathre, R. *et al.* Life-cycle net energy assessment of large-scale hydrogen production via photoelectrochemical water splitting. *Energy Environ. Sci.* **7**, 3264–3278 (2014).

64 Jouny, M., Luc, W. & Jiao, F. General techno-economic analysis of CO_2_ electrolysis systems. *Ind. Eng. Chem. Res.* **57**, 2165–2177 (2018).

65 Bushuyev, O. S. *et al.* What Should We Make with CO2 and How Can We Make It? *Joule* **2**, 825-832 (2018).

66 Li, X. *et al.* Greenhouse gas emissions, energy efficiency, and cost of synthetic fuel production using electrochemical CO_2_ conversion and the fischer–tropsch process. *Energy Fuels* **30**, 5980–5989 (2016).

67 Shaner, M. R., Atwater, H. A., Lewis, N. S. & McFarland, E. W. A comparative technoeconomic analysis of renewable hydrogen production using solar energy. *Energy Environ. Sci.* **9**, 2354–2371 (2016).

68 Colella, W. G., James, B. & Moton, J. M. *Hydrogen Pathways Analysis for Polymer Electrolyte Membrane (PEM) Electrolysis*. (Strategic Analysis Inc, 2014).

69 Verma, S., Kim, B., Jhong, H. R. M., Ma, S. & Kenis, P. J. A. A gross‐margin model for defining technoeconomic benchmarks in the electroreduction of CO_2_. *ChemSusChem* **9**, 1972–1979 (2016).

70 Palmer, C., Saadi, F. & McFarland, E. W. Technoeconomics of commodity chemical production using sunlight. *ACS Sustain. Chem. Eng.* **6**, 7003–7009 (2018).

71 Seider, W. D., Seader, J. D. & Lewin, D. R. *PRODUCT & PROCESS DESIGN PRINCIPLES: SYNTHESIS, ANALYSIS AND EVALUATION, (With CD)*. (John Wiley & Sons, 2009).

72 Neyerlin, K. C., Gu, W., Jorne, J. & Gasteiger, H. A. Study of the Exchange Current Density for the Hydrogen Oxidation and Evolution Reactions. *Journal of The Electrochemical Society* **154**, B631-B635 (2007).

73 Vesborg, P. C. K., Seger, B. & Chorkendorff, I. Recent Development in Hydrogen Evolution Reaction Catalysts and Their Practical Implementation. *The Journal of Physical Chemistry Letters* **6**, 951-957 (2015).

74 Popczun, E. J., Read, C. G., Roske, C. W., Lewis, N. S. & Schaak, R. E. Highly Active Electrocatalysis of the Hydrogen Evolution Reaction by Cobalt Phosphide Nanoparticles. *Angewandte Chemie International Edition* **53**, 5427-5430 (2014).

75 Mahmood, N. *et al.* Electrocatalysts for Hydrogen Evolution in Alkaline Electrolytes: Mechanisms, Challenges, and Prospective Solutions. *Advanced Science* **5**, 1700464 (2018).

76 Satyapal, S. Hydrogen and Fuel Cell Overview (accessed; https://www.energy.gov/sites/prod/files/2017/06/f34/fcto-h2-fc-overview-dla-worldwide-energy-conf-2017-satyapal.pdf.

77 Hernández, S. *et al.* Syngas production from electrochemical reduction of CO2: current status and prospective implementation. *Green Chemistry* **19**, 2326-2346 (2017).

78 Delacourt, C., Ridgway, P. L., Kerr, J. B. & Newman, J. Design of an Electrochemical Cell Making Syngas  ( CO + H_2_ )  from CO_2_ and H_2_O Reduction at Room Temperature. *Journal of The Electrochemical Society* **155**, B42-B49 (2008).

79 Sastre, F. *et al.* Efficient Electrochemical Production of Syngas from CO_2_ and H_2_O by using a Nanostructured Ag/g-C_3_N_4_ Catalyst. *ChemElectroChem* **3**, 1497-1502 (2016).

80 Huynh, M., Shi, C., Billinge, S. J. L. & Nocera, D. G. Nature of Activated Manganese Oxide for Oxygen Evolution. *Journal of the American Chemical Society* **137**, 14887-14904 (2015).

81 Kim, C. *et al.* Insight into Electrochemical CO2 Reduction on Surface-Molecule-Mediated Ag Nanoparticles. *ACS Catalysis* **7**, 779-785 (2017).

82 Materials, D.; https://dioxidematerials.com/technology/co2-electrolysis/.

83 QYResearch. Global Carbon Monoxide Market Research Report 2017 (accessed; https://www.researchmoz.us/global-carbon-monoxide-market-research-report-2017-report.html.

84 Huo, S. *et al.* Coupled Metal/Oxide Catalysts with Tunable Product Selectivity for Electrocatalytic CO2 Reduction. *ACS Applied Materials & Interfaces* **9**, 28519-28526 (2017).

85 Zhang, S., Kang, P. & Meyer, T. J. Nanostructured Tin Catalysts for Selective Electrochemical Reduction of Carbon Dioxide to Formate. *Journal of the American Chemical Society* **136**, 1734-1737 (2014).

86 Materials, D. CO_2_ conversion to formic acid (accessed; https://dioxidematerials.com/technology/formic-acid/.

87 Kuhl, K. P., Cave, E. R., Abram, D. N. & Jaramillo, T. F. New insights into the electrochemical reduction of carbon dioxide on metallic copper surfaces. *Energy & Environmental Science* **5**, 7050-7059 (2012).

88 Institute, M.; https://www.methanol.org/.

89 Administration, U. S. E. I. INTERNATIONAL ENERGY OUTLOOK 2017 (accessed; https://www.eia.gov/outlooks/ieo/.

90 Dinh, C.-T. *et al.* CO_2_ electroreduction to ethylene via hydroxide-mediated copper catalysis at an abrupt interface. *Science* **360**, 783-787 (2018).

91 Mistry, H. *et al.* Highly selective plasma-activated copper catalysts for carbon dioxide reduction to ethylene. *Nature Communications* **7**, 12123 (2016).

92 Markit, I. Ethylene - Global (accessed; https://cdn.ihs.com/www/pdf/Steve-Lewandowski-Big-Changes-Ahead-for-Ethylene-Implications-for-Asia.pdf.

93 Kim, D., Kley, C. S., Li, Y. & Yang, P. Copper nanoparticle ensembles for selective electroreduction of CO_2_ to C_2_–C_3_ products. *Proceedings of the National Academy of Sciences* **114**, 10560-10565 (2017).

94 Lee, S., Park, G. & Lee, J. Importance of Ag–Cu Biphasic Boundaries for Selective Electrochemical Reduction of CO_2_ to Ethanol. *ACS Catalysis* **7**, 8594-8604 (2017).

95 Energy, U. S. D. o. Alternative Fuels Data Center (accessed; https://afdc.energy.gov/data/.

96 MarketsAndMarkets. Propanol Market worth $4.2 billion by 2023 (accessed; https://www.marketsandmarkets.com/PressReleases/isopropyl-alcohol-and-n-propanol.asp.

97 Intelligence, M. Acetaldehyde Market - Segmented by Raw Materials, End-use Applications, and Geography - Growth, Trends, and Forecast (2019 - 2024) (accessed; https://www.mordorintelligence.com/industry-reports/acetaldehyde-market.

98 Research, G. V. Glyoxal Market Size Projected To Reach $2.60 Billion By 2024 (accessed; https://www.grandviewresearch.com/press-release/global-glyoxal-market.

99 Research, H. Acetone Market Size And Forecast, By Application (Solvent, MMA, BPA), By Region (North America, Europe, APAC, Central & South America, MEA) And Trend Analysis, 2014 - 2024 (accessed; https://www.hexaresearch.com/research-report/acetone-market.

100 Research, Z. M. Acetic Acid Market: Massive Vinegar Demand & Related Health Benefits To Spur Growth (accessed; https://www.zionmarketresearch.com/news/global-acetic-acid-market.

101 MEGlobal. Ethylene Glycol Product Guide (accessed; http://www.meglobal.biz/media/product_guides/MEGlobal_MEG.pdf.

102 Suen, N.-T. *et al.* Electrocatalysis for the oxygen evolution reaction: recent development and future perspectives. *Chemical Society Reviews* **46**, 337-365 (2017).

103 ICIS. More oxygen capacity needed to meet energy demand - Air Products (accessed; https://www.icis.com/explore/resources/news/2010/03/16/9343328/more-oxygen-capacity-needed-to-meet-energy-demand-air-products/.

104 Perry, S. C. *et al.* Electrochemical synthesis of hydrogen peroxide from water and oxygen. *Nature Reviews Chemistry* **3**, 442-458 (2019).

105 Siahrostami, S. *et al.* Enabling direct H2O2 production through rational electrocatalyst design. *Nature Materials* **12**, 1137 (2013).

106 Lai, S. C. S. & Koper, M. T. M. Electro-oxidation of ethanol and acetaldehyde on platinum single-crystal electrodes. *Faraday Discussions* **140**, 399-416 (2009).

107 Colmati, F. *et al.* Surface structure effects on the electrochemical oxidation of ethanol on platinum single crystal electrodes. *Faraday Discussions* **140**, 379-397 (2009).

108 Research, Z. M. Acetic Acid Market: Massive Vinegar Demand & Related Health Benefits To Spur Growth (accessed; https://www.zionmarketresearch.com/news/global-acetic-acid-market.

109 Dai, L. *et al.* Electrochemical Partial Reforming of Ethanol into Ethyl Acetate Using Ultrathin Co3O4 Nanosheets as a Highly Selective Anode Catalyst. *ACS Central Science* **2**, 538-544 (2016).

110 Winiwarter, A. *et al.* Towards an atomistic understanding of electrocatalytic partial hydrocarbon oxidation: propene on palladium. *Energy & Environmental Science* **12**, 1055-1067 (2019).

111 Chadderdon, D. J. *et al.* Selective Oxidation of 1,2-Propanediol in Alkaline Anion-Exchange Membrane Electrocatalytic Flow Reactors: Experimental and DFT Investigations. *ACS Catalysis* **5**, 6926-6936 (2015).

112 Jow, J.-J., Lee, A.-c. & Chou, T.-C. Paired electro-oxidation. I. Production of benzaldehyde. *Journal of Applied Electrochemistry* **17**, 753-759 (1987).

113 Zhao, G. *et al.* Electro-oxidation of Benzyl Alcohol in a Biphasic System Consisting of Supercritical CO2 and Ionic Liquids. *The Journal of Physical Chemistry B* **108**, 13052-13057 (2004).

114 Zhu, Y. *et al.* STEP organic synthesis: an efficient solar, electrochemical process for the synthesis of benzoic acid. *Green Chemistry* **16**, 4758-4766 (2014).

115 Insights, G. M. Benzoic Acid Market Size, Industry Analysis Report, Regional Outlook, Application Development, Price Trend, Competitive Market Share & Forecast, 2019 - 2025 (accessed; https://www.gminsights.com/industry-analysis/benzoic-acid-market.

116 Parpot, P., Bettencourt, A. P., Chamoulaud, G., Kokoh, K. B. & Belgsir, E. M. Electrochemical investigations of the oxidation–reduction of furfural in aqueous medium: Application to electrosynthesis. *Electrochimica Acta* **49**, 397-403 (2004).

117 Douthwaite, M. *et al.* The controlled catalytic oxidation of furfural to furoic acid using AuPd/Mg(OH)2. *Catalysis Science & Technology* **7**, 5284-5293 (2017).

118 Kubota, S. R. & Choi, K.-S. Electrochemical Oxidation of 5-Hydroxymethylfurfural to 2,5-Furandicarboxylic Acid (FDCA) in Acidic Media Enabling Spontaneous FDCA Separation. *ChemSusChem* **11**, 2138-2145 (2018).

119 Liu, W.-J. *et al.* Electrochemical Oxidation of 5-Hydroxymethylfurfural with NiFe Layered Double Hydroxide (LDH) Nanosheet Catalysts. *ACS Catalysis* **8**, 5533-5541 (2018).

120 Li, T., Cao, Y., He, J. & Berlinguette, C. P. Electrolytic CO2 Reduction in Tandem with Oxidative Organic Chemistry. *ACS Central Science* **3**, 778-783 (2017).

121 Sugimoto, H., Tsukube, H. & Tanaka, K. Immobilization of a High-Valent Rhenium Complex on an Indium-Doped Tin-Oxide Electrode: Enhanced Catalytic Activity of a trans-Dioxorhenium(V) Complex in Electrochemical Oxidation of Alcohols. *European Journal of Inorganic Chemistry* **2004**, 4550-4553 (2004).

122 van Drunen, J., Napporn, T. W., Kokoh, B. & Jerkiewicz, G. Electrochemical oxidation of isopropanol using a nickel foam electrode. *Journal of Electroanalytical Chemistry* **716**, 120-128 (2014).

123 Research, H. Acetone Market Size And Forecast, By Application (Solvent, MMA, BPA), By Region (North America, Europe, APAC, Central & South America, MEA) And Trend Analysis, 2014 - 2024 (accessed; https://www.hexaresearch.com/research-report/acetone-market.

124 Lyalin, B. V. & Petrosyan, V. A. Oxidation of organic compounds on NiOOH electrode. *Russian Journal of Electrochemistry* **46**, 1199-1214 (2010).

125 Childers, C. L., Huang, H. & Korzeniewski, C. Formaldehyde Yields from Methanol Electrochemical Oxidation on Carbon-Supported Platinum Catalysts. *Langmuir* **15**, 786-789 (1999).

126 Bergamaski, K., Pinheiro, A. L. N., Teixeira-Neto, E. & Nart, F. C. Nanoparticle Size Effects on Methanol Electrochemical Oxidation on Carbon Supported Platinum Catalysts. *The Journal of Physical Chemistry B* **110**, 19271-19279 (2006).

127 Ozoemena, K. I. Nanostructured platinum-free electrocatalysts in alkaline direct alcohol fuel cells: catalyst design, principles and applications. *RSC Advances* **6**, 89523-89550 (2016).

128 Madden, T. H. & Stuve, E. M. II. Mechanisms of Elevated Temperature Methanol Electro-oxidation and Poisoning on Pt/C-Nafion Catalyst Layers. *Journal of The Electrochemical Society* **150**, E571-E577 (2003).

129 Xin, L., Zhang, Z., Qi, J., Chadderdon, D. & Li, W. Electrocatalytic oxidation of ethylene glycol (EG) on supported Pt and Au catalysts in alkaline media: Reaction pathway investigation in three-electrode cell and fuel cell reactors. *Applied Catalysis B: Environmental* **125**, 85-94 (2012).

130 Shaner, M. R., Atwater, H. A., Lewis, N. S. & McFarland, E. W. A comparative technoeconomic analysis of renewable hydrogen production using solar energy. *Energy & Environmental Science* **9**, 2354-2371 (2016).

131 Jouny, M., Luc, W. & Jiao, F. General Techno-Economic Analysis of CO2 Electrolysis Systems. *Industrial & Engineering Chemistry Research* **57**, 2165-2177 (2018).

132 Nurmi, L. *et al.* *From Biomass to Value-Added Furan-Based Platform Chemicals: FURCHEM and CatBio Roadmap. (VTT Technology; No. 326)* (VTT Technical Research Centre of Finland, 2018).

133 Albo, J. & Irabien, A. Cu2O-loaded gas diffusion electrodes for the continuous electrochemical reduction of CO2 to methanol. *Journal of Catalysis* **343**, 232-239 (2016).

134 Anonymous. Methanol. *ICIS Chemical Business* **295**, 36 (2019).

135 Kläusli, T. AVA Biochem: commercialising renewable platform chemical 5-HMF. *Green Process. Synth.* **3**, 235–236 (2014).

136 Dinh, C.-T. *et al.* CO<sub>2</sub> electroreduction to ethylene via hydroxide-mediated copper catalysis at an abrupt interface. *Science* **360**, 783-787 (2018).

137 Waldheim, J. US ethylene slides amid China tariffs. *ICIS Chemical Business* **293**, 19 (2018).

138 Kim, D., Kley, C. S., Li, Y. & Yang, P. Copper nanoparticle ensembles for selective electroreduction of CO<sub>2</sub> to C<sub>2</sub>–C<sub>3</sub> products. *Proceedings of the National Academy of Sciences* **114**, 10560-10565 (2017).

139 Pennington, C. Ethanol. *ICIS Chemical Business* **294**, 30 (2018).

140 Commoprices, Price reports on Ethanal - [acetaldehyde] (accessed 20, March, 2019); https://commoprices.com/en/c/Chemicals/Aldehydes/Ethanal/nc8_29121200?currency=USD&period=Q).

141 Vitasari, C. R., Meindersma, G. W. & de Haan, A. B. Conceptual process design of an integrated bio-based acetic acid, glycolaldehyde, and acetol production in a pyrolysis oil-based biorefinery. *Chemical Engineering Research and Design* **95**, 133-143 (2015).

142 Dietrich, J. Margins in spotlight for US acetone June talks. *ICIS Chemical Business* **293**, 19 (2018).

143 Kelley, L. Acetic acid. *ICIS Chemical Business* **294**, 32 (2018).

144 Commoprices, Price reports on Allyl alcohol (accessed 15, June, 2018); https://commoprices.com/en/c/Chemicals/Acyclic-alcohols-and-their-derivatives/Allyl-alcohol/nc8_29052910?currency=USD&unit=4.

145 Zauba, Import Data and Price of propionaldehyde under HS Code 29121990 (accessed 20, March, 2019); https://www.zauba.com/import-propionaldehyde/hs-code-29121990/p-1-hs-code.html.

146 Tamura, J. *et al.* Electrochemical reduction of CO2 to ethylene glycol on imidazolium ion-terminated self-assembly monolayer-modified Au electrodes in an aqueous solution. *Physical Chemistry Chemical Physics* **17**, 26072-26078 (2015).

147 Hurley, M. Europe MEG demand to grow. *ICIS Chemical Business* **293**, 9 (2018).

148 Tahir, M. *et al.* Electrocatalytic oxygen evolution reaction for energy conversion and storage: A comprehensive review. *Nano Energy* **37**, 136-157 (2017).

149 Song, F. & Hu, X. Exfoliation of layered double hydroxides for enhanced oxygen evolution catalysis. *Nat Commun* **5**, 4477 (2014).

150 Dorris, C. C., Lu, E., Park, S. & Toro, F. H. High-purity oxygen production using mixed ionic-electronic conducting sorbents. (2016).

151 Fuku, K., Miyase, Y., Miseki, Y., Gunji, T. & Sayama, K. Enhanced Oxidative Hydrogen Peroxide Production on Conducting Glass Anodes Modified with Metal Oxides. *ChemistrySelect* **1**, 5721-5726 (2016).

152 Shi, X. *et al.* Understanding activity trends in electrochemical water oxidation to form hydrogen peroxide. *Nat Commun* **8**, 701 (2017).

153 Intratec Solutions, Hydrogen Peroxide Price History (accessed 20, March, 2019); https://www.intratec.us/chemical-markets/hydrogen-peroxide-price.

154 Zhang, Y. *et al.* A novel and effective strategy for electro-oxidation of ethanol to acetaldehyde. *Catalysis Communications* **86**, 119-123 (2016).

155 Juodeikiene, G. *et al.* Green metrics for sustainability of biobased lactic acid from starchy biomass vs chemical synthesis. *Catalysis Today* **239**, 11-16 (2015).

156 Chen, G.-F., Luo, Y., Ding, L.-X. & Wang, H. Low-Voltage Electrolytic Hydrogen Production Derived from Efficient Water and Ethanol Oxidation on Fluorine-Modified FeOOH Anode. *ACS Catalysis* **8**, 526-530 (2017).

157 Dai, L. *et al.* Electrochemical Partial Reforming of Ethanol into Ethyl Acetate Using Ultrathin Co3O4 Nanosheets as a Highly Selective Anode Catalyst. *ACS Cent Sci* **2**, 538-544 (2016).

158 Anonymous. Ethyl acetate. *ICIS Chemical Business* **294**, 45 (2018).

159 Mahmoudian, J. *et al.* Electrochemical Coproduction of Acrylate and Hydrogen from 1,3-Propandiol. *ACS Sustainable Chemistry & Engineering* **5**, 6090-6098 (2017).

160 Anonymous. Acrylic acid. *ICIS Chemical Business* **294**, 36 (2018).

161 Dai, C. *et al.* Electrochemical production of lactic acid from glycerol oxidation catalyzed by AuPt nanoparticles. *Journal of Catalysis* **356**, 14-21 (2017).

162 Zheng, J. *et al.* Hierarchical Porous NC@CuCo Nitride Nanosheet Networks: Highly Efficient Bifunctional Electrocatalyst for Overall Water Splitting and Selective Electrooxidation of Benzyl Alcohol. *Advanced Functional Materials* **27** (2017).

163 Zauba, Export Data and Price of Benzaldehyde under HS Code 29122100 (accessed 30, March, 2019); https://www.zauba.com/export-Benzaldehyde/hs-code-29122100/p-1-hs-code.html.

164 CnAgri-China Agriculture Consultant, Benzoic Acid Exports in Oct. 2013 - Analysis and Viewpoints of China Agriculture (accessed 20, March, 2019); http://en.cnagri.com/news/insight/20140107/298057.html.

165 You, B., Liu, X., Jiang, N. & Sun, Y. A General Strategy for Decoupled Hydrogen Production from Water Splitting by Integrating Oxidative Biomass Valorization. *J Am Chem Soc* **138**, 13639-13646 (2016).

166 Zauba, Export Data and Price of 2 furoic acid under HS Code 29321910 (accessed 20, March, 2019); https://www.zauba.com/export-2+FUROIC+ACID/hs-code-29321910/p-1-hs-code.html.

167 Jiang, N. *et al.* Electrocatalysis of Furfural Oxidation Coupled with H2

Evolution via Nickel-Based Electrocatalysts in Water. *ChemNanoMat* **3**, 491-495 (2017).

168 Zauba, Export Data and Price of 2 5 furandicarboxylic acid under HS Code 29321990 (accessed 20, March, 2019); https://www.zauba.com/export-2+5+furandicarboxylic+acid/hs-code-29321990/p-1-hs-code.html.

169 Wu, T. *et al.* Vapor-phase hydrothermal growth of single crystalline NiS2 nanostructure film on carbon fiber cloth for electrocatalytic oxidation of alcohols to ketones and simultaneous H2 evolution. *Nano Research* **11**, 1004-1017 (2017).

170 Liu, Y. *et al.* Electrooxidation of Ethanol and Methanol Using the Molecular Catalyst [{Ru4O4(OH)2(H2O)4}(gamma-SiW10O36)2](10.). *J Am Chem Soc* **138**, 2617-2628 (2016).

171 Kirschner, M. Formaldehyde. *ICIS Chemical Business Americas* **271**, 34 (2007).

172 Chen, Y. X., Miki, A., Ye, S., Sakai, H. & Osawa, M. Formate, an Active Intermediate for Direct Oxidation of Methanol on Pt Electrode. *J Am Chem Soc* **125** (2003).

173 Chen, Y. X. *et al.* Nanotechnology makes biomass electrolysis more energy efficient than water electrolysis. *Nat Commun* **5**, 4036 (2014).

174 PharmaCompass, glycolic acid price (accessed 20, March, 2019); https://www.pharmacompass.com/price/glycolic-acid.

175 Matsumoto, T. *et al.* CO2-free power generation on an iron group nanoalloy catalyst via selective oxidation of ethylene glycol to oxalic acid in alkaline media. *Sci Rep* **4**, 5620 (2014).

176 Reuters, Global Oxalic Acid Market Projected to grow at a CAGR of 4% during 2018-2025 (accessed 20, March, 2019); https://www.reuters.com/brandfeatures/venture-capital/article?id=39503.

177 Joback, K. G. & Reid, R. C. ESTIMATION OF PURE-COMPONENT PROPERTIES FROM GROUP-CONTRIBUTIONS. *Chemical Engineering Communications* **57**, 233-243 (1987).

178 Ilas, A., Ralon, P., Rodriguez, A. & Taylor, M. Renewable power generation costs in 2017. *International Renewable Energy Agency (IRENA): Abu Dhabi, UAE* (2018).

179 Alibaba, Potassium Bicarbonate Price, Wholesale & Suppliers (accessed 15, March, 2019); https://www.alibaba.com/showroom/potassium-bicarbonate-price.html.

180 Zoelle, A. *et al.* Cost and Performance Baseline for Fossil Energy Plants Volume 1a: Bituminous Coal (PC) and Natural Gas to Electricity Revision 3. Report No. DOE/NETL-2015/1723 United States 10.2172/1480987 NETL English, Medium: ED (; NETL, 2015).

181 InfoMine, Mining Intelligence and Technology (accessed 22, January, 2019); http://www.infomine.com.

182 Vanadium Price, vanadium, vanadium price, vanadium pentoxide, ferro vanadium (accessed 22, January, 2019); https://www.vanadiumprice.com/.

183 NREL, Solar Data (accessed 22, March, 2019); https://www.nrel.gov/gis/data-solar.html.

184 Molel, E., Phillips, H. & Smith, A. 1, 3-Propanediol from crude glycerol. (2015).

185 Yanelli, A. MPG. *ICIS Chemical Business* **293**, 34 (2018).

186 Intratec Solutions, Glycerol Price History & Forecast (accessed 21, March, 2019); https://www.intratec.us/chemical-markets/glycerol-price.

187 Commoprices, Price reports on Benzyl alcohol (accessed 15, June, 2018); https://commoprices.com/en/c/Chemicals/Acyclic-alcohols-and-their-derivatives/Benzyl-alcohol/nc8_29062100?currency=USD.

188 Commoprices, Price reports on Furfural (accessed 15, June, 2018); https://commoprices.com/en/c/Chemicals/Heterocyclic-oxygen-heteroatom/Furfural/nc8_29321200?currency=USD&period=Q.

189 Nurmi, L. *et al.* From biomass to value-added furan-based platform chemicals: FURCHEM and CatBio roadmap. (2018).

190 Kazi, F. K., Patel, A. D., Serrano-Ruiz, J. C., Dumesic, J. A. & Anex, R. P. Techno-economic analysis of dimethylfuran (DMF) and hydroxymethylfurfural (HMF) production from pure fructose in catalytic processes. *Chemical Engineering Journal* **169**, 329-338 (2011).

191 Anonymous. Isopropanol. *ICIS Chemical Business* **294**, 34 (2018).

192 S., A. A., Yumei, Z., Davion, H. & Narasi, S. The Electrochemical Reduction of Carbon Dioxide to Formate/Formic Acid: Engineering and Economic Feasibility. *ChemSusChem* **4**, 1301-1310 (2011).

193 Sathre, R. *et al.* Life-cycle net energy assessment of large-scale hydrogen production via photoelectrochemical water splitting. *Energy & Environmental Science* **7**, 3264-3278 (2014).

194 Pérez-Fortes, M., Schöneberger, J. C., Boulamanti, A., Harrison, G. & Tzimas, E. Formic acid synthesis using CO2 as raw material: Techno-economic and environmental evaluation and market potential. *International Journal of Hydrogen Energy* **41**, 16444-16462 (2016).

195 Palmer, C., Saadi, F. & McFarland, E. W. Technoeconomics of Commodity Chemical Production Using Sunlight. *ACS Sustainable Chemistry & Engineering* **6**, 7003-7009 (2018).

196 Spurgeon, J. M. & Kumar, B. A comparative technoeconomic analysis of pathways for commercial electrochemical CO2 reduction to liquid products. *Energy & Environmental Science* **11**, 1536-1551 (2018).

197 Häussinger, P., Lohmüller, R. & Watson, A. M. in *Ullmann's Encyclopedia of Industrial Chemistry* (Wiley-VCH Verlag GmbH & Co. KGaA, Weinheim, Germany, 2011).

198 Häussinger, P., Lohmüller, R. & Watson, A. M. in *Ullmann's Encyclopedia of Industrial Chemistry* (Wiley-VCH Verlag GmbH & Co. KGaA, Weinheim, Germany, 2011).

199 Staffell, I. *et al.* The role of hydrogen and fuel cells in the global energy system. *Energy and Environmental Science* **12**, 463-491 (2019).

200 George, C. in *Kirk-Othmer Encyclopedia of Chemical Technology* (John Wiley & Sons, Inc., Hoboken, NJ, USA, 2001).

201 QYResearch, Global Carbon Monoxide Market Research Report 2017, Trends, Share, Size Research Report (accessed 16, April, 2019); https://www.qyresearchreports.com/report/global-carbon-monoxide-market-research-report-2017.htm.

202 Hietala, J. *et al.* in *Ullmann's Encyclopedia of Industrial Chemistry* 1-22 (Wiley-VCH Verlag GmbH & Co. KGaA, Weinheim, Germany, 2016).

203 Ott, J. *et al.* in *Ullmann's Encyclopedia of Industrial Chemistry* (Wiley-VCH Verlag GmbH & Co. KGaA, Weinheim, Germany, 2012).

204 Mears, D. E. & Eastman, A. D. in *Kirk-Othmer Encyclopedia of Chemical Technology* (John Wiley & Sons, Inc., Hoboken, NJ, USA, 2005).

205 Zimmermann, H. & Walzl, R. in *Ullmann's Encyclopedia of Industrial Chemistry* (Wiley-VCH Verlag GmbH & Co. KGaA, Weinheim, Germany, 2009).

206 Kosaric, N. *et al.* in *Ullmann's Encyclopedia of Industrial Chemistry* 1-72 (Wiley-VCH Verlag GmbH & Co. KGaA, Weinheim, Germany, 2011).

207 Klabunde, J., Bischoff, C. & Papa, A. J. in *Ullmann's Encyclopedia of Industrial Chemistry* 1-14 (Wiley-VCH Verlag GmbH & Co. KGaA, Weinheim, Germany, 2018).

208 Hagemeyer, H. J. & Staff, U. b. in *Kirk-Othmer Encyclopedia of Chemical Technology* 1-16 (John Wiley & Sons, Inc., Hoboken, NJ, USA, 2014).

209 Eckert, M., Fleischmann, G., Jira, R., Bolt, H. M. & Golka, K. in *Ullmann's Encyclopedia of Industrial Chemistry* (Wiley-VCH Verlag GmbH & Co. KGaA, Weinheim, Germany, 2006).

210 Mohamad, M. H. *et al.* A Review of Acetol: Application and Production. *American Journal of Applied Sciences* **8**, 1135-1139 (2011).

211 Weber, M., Weber, M. & Kleine-Boymann, M. in *Ullmann's Encyclopedia of Industrial Chemistry* (Wiley-VCH Verlag GmbH & Co. KGaA, Weinheim, Germany, 2004).

212 Sullivan, C. J., Kuenz, A. & Vorlop, K.-D. in *Ullmann's Encyclopedia of Industrial Chemistry* 1-15 (Wiley-VCH Verlag GmbH & Co. KGaA, Weinheim, Germany, 2018).

213 Hort, E. V. & Taylor, P. in *Kirk-Othmer Encyclopedia of Chemical Technology* (John Wiley & Sons, Inc., Hoboken, NJ, USA, 2003).

214 Weber, M., Pompetzki, W., Bonmann, R. & Weber, M. in *Ullmann's Encyclopedia of Industrial Chemistry* 1-19 (Wiley-VCH Verlag GmbH & Co. KGaA, Weinheim, Germany, 2014).

215 Wagner, F. S. & Staff, U. b. in *Kirk-Othmer Encyclopedia of Chemical Technology* 1-21 (John Wiley & Sons, Inc., Hoboken, NJ, USA, 2014).

216 Nagato, N. in *Kirk-Othmer Encyclopedia of Chemical Technology* (John Wiley & Sons, Inc., Hoboken, NJ, USA, 2004).

217 Sánchez, G. *et al.* The effect of catalyst modification on the conversion of glycerol to allyl alcohol. *Applied Catalysis B: Environmental* **152-153**, 117-128 (2014).

218 Xu, J. *et al.* Utilization of bio-based glycolaldehyde aqueous solution in organic synthesis: application to the synthesis of 2,3-dihydrofurans. *Green Chemistry* (2019).

219 UKEDA, H., ISHII, T., SAWAMURA, M. & ISOBE, K. Glycolaldehyde Production from Ethylene Glycol with Immobilized Alcohol Oxidase and Catalase. *Bioscience, Biotechnology, and Biochemistry* **62**, 1589-1591 (1998).

220 Hensel, A. in *Ullmann's Encyclopedia of Industrial Chemistry* 1-8 (Wiley-VCH Verlag GmbH & Co. KGaA, Weinheim, Germany, 2018).

221 Forkner, M. W. *et al.* in *Kirk-Othmer Encyclopedia of Chemical Technology* (John Wiley & Sons, Inc., Hoboken, NJ, USA, 2004).

222 Yue, H., Zhao, Y., Ma, X. & Gong, J. Ethylene glycol: properties, synthesis, and applications. *Chemical Society Reviews* **41**, 4218 (2012).

223 Kirschner, M. J. *et al.* in *Ullmann's Encyclopedia of Industrial Chemistry* 1-32 (Wiley-VCH Verlag GmbH & Co. KGaA, Weinheim, Germany, 2017).

224 Abdollahi, M. & Hosseini, A. in *Encyclopedia of Toxicology: Third Edition* 967-970 (Wiley-VCH Verlag GmbH & Co. KGaA, Weinheim, Germany, 2014).

225 Siahrostami, S. *et al.* Enabling direct H2O2 production through rational electrocatalyst design. *Nature Materials* **12**, 1137-1143 (2013).

226 Le Berre, C., Serp, P., Kalck, P. & Torrence, G. P. in *Ullmann's Encyclopedia of Industrial Chemistry* 1-34 (Wiley-VCH Verlag GmbH & Co. KGaA, Weinheim, Germany, 2014).

227 Guy, A., J.R. Ethyl acetate. *ICIS Chemical Business* **285**, 35 (2014).

228 Ohara, T. *et al.* in *Ullmann's Encyclopedia of Industrial Chemistry* (Wiley-VCH Verlag GmbH & Co. KGaA, Weinheim, Germany, 2011).

229 Starr, J. N. & Westhoff, G. in *Ullmann's Encyclopedia of Industrial Chemistry* 1-8 (Wiley-VCH Verlag GmbH & Co. KGaA, Weinheim, Germany, 2014).

230 Brühne, F. & Wright, E. in *Ullmann's Encyclopedia of Industrial Chemistry* (Wiley-VCH Verlag GmbH & Co. KGaA, Weinheim, Germany, 2011).

231 Opgrande, J. L., Brown, E. E., Hesser, M. & Andrews, J. in *Kirk-Othmer Encyclopedia of Chemical Technology* (John Wiley & Sons, Inc., Hoboken, NJ, USA, 2003).

232 Eseyin, A., E., Steele, P., H. & Philip, H. S. An overview of the applications of furfural and its derivatives. *International Journal of Advanced Chemistry* **3**, 42 (2015).

233 Hoydonckx, H. E., Van Rhijn, W. M., Van Rhijn, W., De Vos, D. E. & Jacobs, P. A. in *Ullmann's Encyclopedia of Industrial Chemistry* (Wiley-VCH Verlag GmbH & Co. KGaA, Weinheim, Germany, 2007).

234 BioConSepT.

235 Franz, A. W. *et al.* in *Ullmann's Encyclopedia of Industrial Chemistry* 1-34 (Wiley-VCH Verlag GmbH & Co. KGaA, Weinheim, Germany, 2016).

236 Miltenberger, K. in *Ullmann's Encyclopedia of Industrial Chemistry* (Wiley-VCH Verlag GmbH & Co. KGaA, Weinheim, Germany, 2000).

237 Riemenschneider, W. & Tanifuji, M. in *Ullmann's Encyclopedia of Industrial Chemistry* (Wiley-VCH Verlag GmbH & Co. KGaA, Weinheim, Germany, 2011).

238 Lazard, Levelized Cost of Energy and Levelized Cost of Storage 2018 (accessed 30 May 2019); https://www.lazard.com/perspective/levelized-cost-of-energy-and-levelized-cost-of-storage-2018/.

239 Colella, W. G., James, B. & Moton, J. M. Hydrogen Pathways Analysis for Polymer Electrolyte Membrane (PEM) Electrolysis. *Strategic Analysis Inc* (2014).
